# Deconvolution of Light‐Induced Ion Migration Phenomena by Statistical Analysis of Cathodoluminescence in Lead Halide‐Based Perovskites

**DOI:** 10.1002/advs.202103729

**Published:** 2022-03-03

**Authors:** Erfan Shirzadi, Nicolas Tappy, Fatemeh Ansari, Mohammad Khaja Nazeeruddin, Anders Hagfeldt, Paul J. Dyson

**Affiliations:** ^1^ Institute of Chemical Sciences and Engineering Swiss Federal Institute of Technology Lausanne (EPFL) Lausanne CH‐1015 Switzerland; ^2^ Laboratoire des Matériaux Semiconducteurs Institute of Materials Faculty of Engineering École Polytechnique Fédérale de Lausanne Lausanne 1015 Switzerland

**Keywords:** cathodoluminescence, ion migrations, perovskites, surface passivation

## Abstract

Studying the compositional instability of mixed ion perovskites under light illumination is important to understand the mechanisms underlying their efficiency and stability. However, current techniques are limited in resolution and are unable to deconvolute minor ion migration phenomena. Here, a method that enables ion migration to be studied allowing different segregation mechanisms to be elucidated is described. Statistical analysis is applied to cathodoluminescence data to generate compositional distribution histograms. Using these histograms, two different ion migration phenomena, horizontal ion migration (HIM) and vertical ion migration (VIM), are identified in different perovskite films. It is found that most passivating agents inhibit HIM, but not VIM. However, VIM can be reduced by deposition of imidazolium iodide on the perovskite surface. This method can be used to study perovskite‐based devices efficiency and stability by providing molecular level mechanistic understanding of passivation approaches leading to performance improvement of perovskite solar cells via rational design.

## Graphical abstract

Light causes ions to migrate two different directions in organic/inorganic lead halide: horizontal and vertical. A method combining cathodoluminescence and statistics is developed that quantifies the different types of ion migrations for both halides and cations in different types of perovskites. A possible mechanism for different modes of ion migration is discussed.

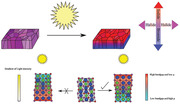

## 1 Introduction

Organic/inorganic lead halide perovskites have been extensively studied as photovoltaic materials in a variety of optoelectronic devices including solar cells, light‐emitting diodes, memory devices, and photodetectors.^[^ ^1^ ^]^ However, due to the polycrystalline nature of these materials and the compositional complexity of various perovskites fabricated from mixed cations and anions, their compositional integrity is prone to change under operational conditions.^[^ ^2^ ^]^ Perovskites have the general chemical formula of ABX_3_, where A is typically a cation such as methylammonium (MA^+^), formamidinium (FA^+^), or cesium (Cs^+^), B is a divalent cation such as Pb^2+^ or Sn^2+^ and X^−^ is a halide. Phase segregation plays an important role in charge carrier recombination and the overall performance of perovskite solar cells (PSCs).^[^ ^3^ ^]^ For instance, ion migration has been suggested to lead to hysteresis in current density–voltage (*I*–*V*) measurements.^[^ ^4^ ^]^ Studies link ion migration to instability and performance loss due to amorphization, phase segregation, and defect formation in perovskite solar cells.^[^ ^5^ ^]^ Therefore, having a tool to monitor ion migration is necessary to develop efficient and stable perovskite solar cells.

Among the different ions which can participate in ion migration, halide ions appear to be particularly mobile due to their low activation energy for ion migration.^[^ ^6^ ^]^ A reversible redshift emission was observed for (CH_3_NH_3_)Pb(Br*_x_* I_1−_ *_x_*)_3_ upon light soaking by Hoke et al.^[^ ^7^ ^]^ A similar observation attributed to photoinduced ion migration in photoluminescence (PL) spectra was reported by Duong et al. for the mixed cation‐anion Rb_0.05_(Cs_0.1_MA_0.15_FA_0.75_)_0.95_PbI_2_Br perovskite.^[^ ^8^ ^]^ In the same study Duong et al. observed a spatial distribution on the perovskite film surface linked to emission shifts in the PL spectra using a micro‐PL scanning approach. In another study, Bischak et al., used cathodoluminescence (CL) to study short‐term light‐induced changes in the halide distribution in MAPb(I_0.1_Br_0.9_)_3_ film. After exposure to a 405 nm LED for 5 min, inhomogeneous I‐rich clusters were observed throughout the film, which were stabilized by polarons.^[^ ^9^ ^]^

Various hypothesis have been suggested for light‐induced halide migration, which can be broadly categorized into three different subgroups.^[^ ^10^ ^]^ First, based on a study by Brivio et al., light causes the kinetic barrier to be overcome such that the metastable mixed perovskite segregates.^[^ ^11^ ^]^ Second, light‐induced structural strains due to polarons promote demixing.^[^ ^12^ ^]^ Third, photogenerated charge carriers induce strain in the perovskite structure and the carrier generation gradient causes light‐induced ion migration.^[^ ^13^ ^]^

Despite many efforts to understand light‐induced ion migration, a tool to quantify and spatially analyze ion migration is missing.^[^ ^14^ ^]^ Current methods to quantify ion migration, such as transient absorption spectroscopy (TA) and X‐ray diffraction (XRD), lack the accuracy to analyze small amounts of ion migration and lack the spatial resolution to analyze at the grain level. In a typical PL measurement, the data are biased by the more photoluminescent perovskite composition. In other words, we cannot tell the typical redshift is due to I‐rich grains becoming more luminescent or due to ion migration.^[^ ^15^ ^]^ A method that can deconvolute the effect of intensity and focus only on the bandgap is needed. Although CL has previously been used to observe the ion migration with high spatial resolution,^[8,12b,16]^ it has not been used quantitatively.^[^ ^14^ ^]^ Here, we present a method to analyze and quantify ion migration in perovskite films based on CL based on a detailed statistical analysis of the data. Based on this analysis, two different ion migration phenomena were identified, i.e., horizontal ion migration (HIM) and vertical ion migration (VIM), which take place independently. Segregation of A‐cations for Cs_0.3_FA_0.7_PbI_3_ films was not observed, confirming that HIM invokes halide vacancies but not A‐cation vacancies. We also show that surface passivating materials inhibit HIM, but not VIM, which should be the focus of future studies to increase the lifetime of perovskite‐based optoelectronic devices.

## 2 Results and Discussion

### 2.1 Cathodoluminescence of Perovskite Films

CL was used to study the compositional distribution of the perovskite films. Charge carriers are generated by the impact of the electron beam with the semiconductor recombined to emit photons corresponding to the bandgap of the semiconductor.^[^ ^14^, ^17^ ^]^ Based on a study by Bischak et al.,^[^ ^18^ ^]^ a 3 keV beam energy ensures the majority of the carrier generation volume is within ≈60 nm depth of the surface with a radius from impact electron beam with a similar value, which is typically smaller than the grain diameter (see Figure S1, Supporting Information). The generated charge carriers from the activation volume travel within the width of perovskite film due to their high diffusion lengths.^[^ ^19^ ^]^ As the recombination rate is high at the interface of the grains, their diffusion volume is mainly limited within the grains.^[^ ^19^, ^20^ ^]^ Therefore, the electrons and holes generated by the electron beam mostly travel within boundaries of grains and the CL signals apparently originate from the impacted grains rather than adjacent grains. In addition, it is necessary to consider reabsorption of emitted photons within the bulk by the surface for direct bandgap semiconductors with high absorption coefficients such as lead halide perovskites. Therefore, if the surface has a lower bandgap than the interior of the bulk, a redshift in the maximum of emission signal will be observed,^[^ ^14^ ^]^ because the CL signal more reflects the surface composition than the interior composition.^[^ ^14^, ^17^, ^21^ ^]^

**Figure 1:**
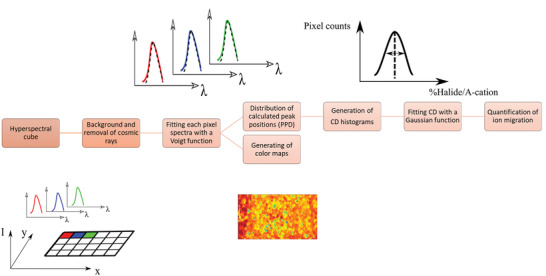
Sequential steps to analyze the CL data. After the acquisition of the CL experiment, the background and cosmic rays are removed from the data. Then each pixel of the spectra is fitted with a Voigt function. The fitting then is used to generate the color maps and generation of PPD histogram. The PPD histograms can then be converted to the CD histograms if a linear relationship between the bandgap and the content of halogen or A‐cation exists. Then the CD histogram is fitted with a gaussian function to quantify the ion migrations.

The perovskite films (Cs_0.08_MA_0.12_FA_0.80_PbI_2.64_Br_0.36_ and Cs_0.3_FA_0.7_PbI_3_) were analyzed by CL electron microscopy with a 3 keV electron beam. A liquid helium‐cooled stage (≈10 K) was used to minimize perovskite beam‐induced decomposition and to increase the luminescence. After the acquisition, the data were analyzed according to the steps shown in **Figure** **1**. The obtained data is in the form of hyperspectral cubic data which contains pixels at the *x*‐ and *y*‐position with a luminescence spectrum corresponding to that point. After removing the background and cosmic rays, the data were treated with a Python script. Each pixel spectrum was fitted with a Voigt function. The parameters of fitted functions such as peak center and full width at half maximum (FWHM) were plotted as separate color maps, i.e., if the center of the fitted Voigt functions (which corresponds to the emission peak maximum of each pixel) is represented in a color (herein red for higher wavelengths and blue for lower wavelengths), it would generate a map of fitted peak centers. These images (color maps for each of Voigt parameters), along with the goodness of fit, their uncertainty and secondary electron images are presented in the supplementary information (Figures S2–S13, Supporting Information). Note that regions of the film, which do not give a good peak fitness are shown in gray and were not considered for further analysis. This way of analyzing the data allow us to focus on peak maximum mainly which corresponds to bandgap without being biased from the intensity of the peak.

**Figure 2:**
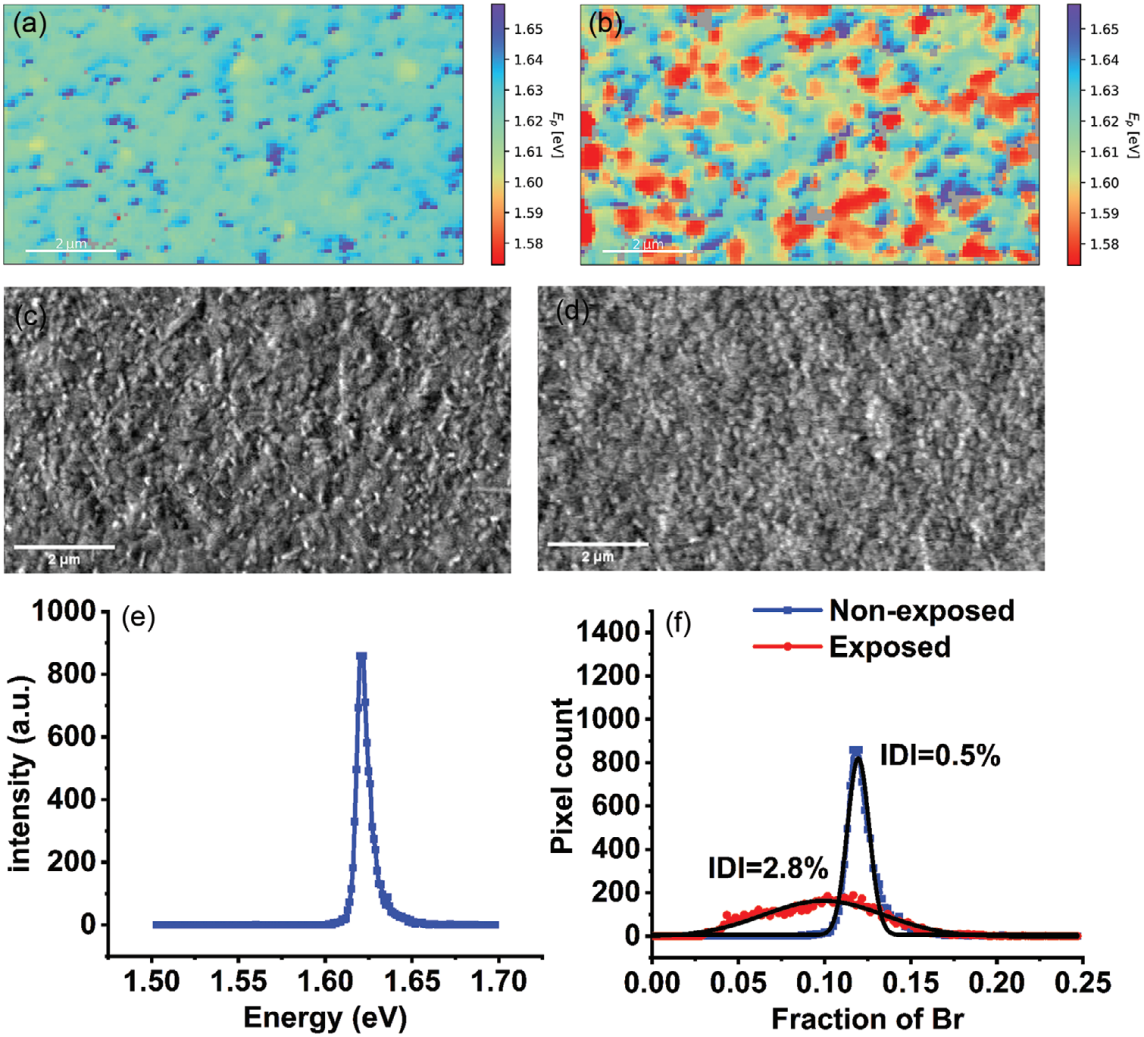
Analysis of non‐illuminated and illuminated Cs_0.08_MA_0.12_FA_0.80_PbI_2.64_Br_0.36_ perovskite. The map of fitted peak positions for a) non‐illuminated Cs_0.08_MA_0.12_FA_0.80_PbI_2.64_Br_0.36_ and b) illuminated Cs_0.08_MA_0.12_FA_0.80_PbI_2.64_Br_0.36_ film. c) The secondary electron image of non‐illuminated and d) illuminated Cs_0.08_MA_0.12_FA_0.80_PbI_2.64_Br_0.36_ film (Scale bar = 2 µm). e) PPD histogram of non‐illuminated region of the film. f) CD histograms for the Cs_0.08_MA_0.12_FA_0.80_PbI_2.64_Br_0.36_ film that show IDI of 0.5 ± 0.01% (*n* = 196, Adj. *R* ^2^ = 0.97) for non‐illuminated and 2.8 ± 0.1% (*n* = 196, Adj. *R* ^2^ = 0.96) for the illuminated film.

By examining the fitted CL data of the perovskite films, it was found that the position of peak centers differs from region to region of the image. As an example, **Figure** **2**a,b shows the color map of peak center position image of a Cs_0.08_MA_0.12_FA_0.80_PbI_2.64_Br_0.36_ film (MA^+^: methylammonium, FA^+^: formamidinium), i.e., some regions of the film were emitting light at higher and some at lower *λ* upon electron impact. The secondary electron images of the films are also provided in Figure 2c,d. For instance, the distribution in a peak position distribution (PPD) in Figure 2a is compiled in a histogram (Figure 2e) where each point on the PPD histogram represents the number of pixels (*Y*) that emits light at particular energy (*X*). The distribution is mainly due to the compositional difference among the various perovskite grains. Despite a homogenous perovskite precursor solution, the distribution shows that the composition of the perovskite films varies slightly in from domain to domain.

Usually the bandgap (presented in energy unit) of mixed perovskites has a linear relationship with the percentage halide used in their fabrication.^[^ ^22^ ^]^ Therefore, the PPD can be converted to a compositional distribution (CD) histogram where the *X*‐axis corresponds to the molar fraction of the halide or A‐site cation and the *Y*‐axis is the population or pixel count. A‐site cations also contribute to the bandgap with a linear relationship, but with a less impact.^[^ ^23^ ^]^ Here, we attribute the variation of the bandgap of the Cs_0.08_MA_0.12_FA_0.80_PbI_2.64_Br_0.36_ perovskite only to the halide content difference between the grains (the impact of A‐site cation migration on the bandgap is discussed later). Assuming that only halides contribute to the variations of the bandgap in different regions of the Cs_0.08_MA_0.12_FA_0.80_PbI_2.64_Br_0.36._ film, the *X*‐axis of the PPD can be converted to the fraction of the bromide using Equation (1)^[^ ^24^ ^]^

(1) $$\mathrm{Bandgap}\left(\mathrm{eV}\right)=0.62x+1.55$$

where *x* is the fraction of bromide relative to the total halide content. The intercept is calculated from the peak position of CD of the non‐exposed film with *x* = 0.12. The CD histogram with its Gaussian fit is presented in Figure 2f as a blue curve. The CD was fitted with a single Gaussian function (the summary of the fits parameters is presented in Table S1, Supporting Information). A small shoulder is usually observed at high energy which corresponds to a small proportion of high bromide content (higher bandgap) perovskite in the film. For simplicity such small deviations are ignored.

### 2.2 Photoinduced Ion Migration in the Cs_0.08_MA_0.12_FA_0.80_PbI_2.64_Br_0.36_ Perovskite

Prior to CL measurements, the perovskite films were exposed to white LED light with an intensity of 100 mW cm^−2^ (1 sun) under a nitrogen atmosphere at ≈37 °C for 12 h. The CL data of the exposed and non‐exposed to light samples were collected from the same film to avoid minor compositional inconsistencies between the films (see the Experimental Section for full measurement details).

As shown in Figure 2f, by comparing CDs of exposed and non‐exposed parts of the perovskite film, two differences were observed. First, the CD of the exposed part has significantly larger FWHM values. The wider CD of the exposed part of the film may be attributed to the formation of Br^−^‐rich and I^−^‐rich domains on the surface of perovskite film due to HIM. Second, the CD fitted peak position shifts toward lower energies (redshift). A shift to lower energy in the CD is also observed following exposure of the film to light (Figure 2b,f). The redshift in the maximum of the fitted Gaussian function of the CD for the exposed perovskite after exposure to light implies that the surface of perovskite has become more I^−^‐rich and therefore the bulk of the film became more Br^−^‐rich and we refer to this type of ion demixing as VIM.

Based on the study by Yoon et al., the amount of VIM can be influenced and biased by considering the low bandgap regions (i.e., iodide rich clusters), which serve as sinks for generated charge carriers following electron beam impact.^[^ ^20a^ ^]^ The majority of the signal comes from the grain which is impacted by the electron beam, as the recombination at the grain boundaries, as discussed earlier, is usually high.^[^ ^25^ ^]^ Additionally, it might be that energy transfer to lower bandgap regions is more pronounced for segregations when the difference in the bandgap between two regions is high. Here, the mean bandgap change corresponds to about 0.01 eV that might not be sufficient for a pronounced bias. In contrast, HIM can also be influenced by VIM if the rate at which ions migrate in the vertical direction is significantly different from grain to grain.

**Figure 3:**
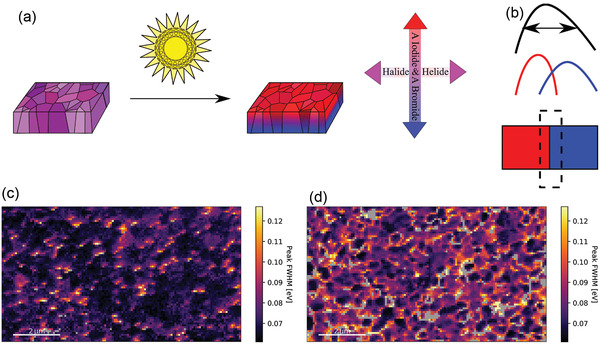
HIM and VIM in perovskite films. a) Schematic presentation of HIM and VIM. Before illumination the perovskite domains have low distribution of composition whereas after exposure to light the surface of perovskite becomes I^−^‐rich and the distribution of composition on the surface becomes wider. c,d) Maps of width of fitted functions for the Cs_0.08_MA_0.12_FA_0.80_PbI_2.64_Br_0.36_ perovskite: non‐exposed and exposed regions, respectively.

As shown in **Figure** **3**a, VIM has a directionality where an iodide‐rich perovskite forms on the surface, whereas HIM causes different domains of the film to have different compositions and thus different bandgaps. The resulting CD histogram can be used to quantify the HIM and VIM ion migration. A wider CD histogram for the exposed films corresponds to light‐induced ion migration which results in a variety of perovskite compositions on the surface of the perovskite film. Therefore, the magnitude of HIM is dependent on the or standard deviation, *σ*, of the Gaussian fitted CD curve and can be quantified for a Gaussian fit using Equation (2).

To quantify how much the actual distribution differs from the nominal composition (the stoichiometric distribution), it is necessary to quantify how many pixels the distribution with *x* ion fraction have which is equal to *dxg*(*x*), where *g*(*x*) = $ae^{\frac{-x^{2}}{2w^{2}}\;}$ is the Gaussian fit for the CD curve assuming *x* = 0 is at the maximum of the curve. However, the ion dispersity is not only proportional to the area but also the value of *x*. The magnitude of ion dispersity is proportional to *xdxg*(*x*). To obtain the ion dispersity related to *x*, it needs to be divided by the total area of the Gaussian function ($a\sqrt{2\pi w^{2}}$) as the ion dispersity index (IDI) corresponds to the portion of ions moved in the total population of ions from the map (picture) where the data are extracted. Thus

(2) $$\mathrm{Ion}\;\mathrm{dispersity}\;\mathrm{index}\;(\mathrm{IDI})=\frac{2a\int_{0}^{+\infty }e^{\frac{-x^{2}}{2w^{2}}\;}xdx}{a\sqrt{2\pi w^{2}}}*100\%\approx 80\;w$$

where *a* is the magnitude and *w* is a parameter related to the FWHM of the Gaussian function (FWHM = $2\sqrt{2\ln2}w$). The IDIs of the Cs_0.08_MA_0.12_FA_0.80_PbI_2.64_Br_0.36_ films are presented in Figure 2f. The amount of HIM is calculated using Equation (3)

(3) $$\mathrm{Magnitude}\;\mathrm{of}\;\mathrm{HIM}=\mathrm{ID}I_{\mathrm{exposed}}-\mathrm{ID}I_{\mathrm{non}-\mathrm{exposed}}$$

which gives 2.3% HIM due to light exposure for 12 h. As 2.3% of ion migration is relatively low, we did not observe two separate peaks in the CD histogram and it can be fitted with a Gaussian function, and a similar argument applies to the rest of the data presented herein. Additionally, ion migration did not result in the formation of only two stoichiometrically defined perovskites, rather it resulted in a gradient of compositions distributed among different grains.

The magnitude of VIM can be estimated from the difference in the peak position of two fitted PPD gaussian curves of exposed and non‐exposed parts, which yields 2.0% VIM.

1% of HIM corresponds to net migration of ions (halides in mixed halide perovskite) in the horizontal direction which causes the bandgap to change, as examples all the following reactions have 10% HIM

(4) $$\begin{array}{c} \mathrm{MAPb}I_{1.5}Br_{1.5}\to 0.8\mathrm{MAPb}I_{1.5}Br_{1.5}+0.1\mathrm{MAPb}I_{3}+0.1\mathrm{MAPbB}r_{3} \end{array}$$

(5) $$\begin{array}{ccc} \mathrm{MAPb}I_{1.5}Br_{1.5} & \to & 0.75\mathrm{MAPb}I_{1.5}Br_{1.5}+0.05\mathrm{MAPb}I_{3}\\ & & +\;0.05\mathrm{MAPbB}r_{3}+0.075\mathrm{MAPb}I_{2}\mathrm{Br}\\ & & +\;0.075\mathrm{MAPbIB}r_{2} \end{array}$$

(6) $$\begin{array}{ccc} \mathrm{MAPb}I_{1.5}{\mathrm{Br}}_{1.5} & \to & 0.7\mathrm{MAPb}I_{1.5}{\mathrm{Br}}_{1.5}+0.05\mathrm{MAPb}I_{3}\\ & & +\;0.05\mathrm{MAPbB}r_{3}+0.05\mathrm{MAPb}I_{2}\mathrm{Br}\\ & & +\;0.05\mathrm{MAPbIB}r_{2}+0.05\mathrm{MAPb}I_{2.5}{\mathrm{Br}}_{0.5}\\ & & +\;0.05\mathrm{MAPb}I_{0.5}{\mathrm{Br}}_{2.5} \end{array}$$

In reality, a gradient of distributions is obtained, and the ones similar to the initial distribution will have a higher population than the ones further from it. Such distribution may be fitted with a Gaussian function and as shown in Equations (2) and (3), which can be used to estimate the HIM.

Having grains with different bandgaps adjacent to each other causes the width of the emission at the grain boundaries to broaden (Figure 3b). Therefore, the FWHM of the CL peaks from each pixel also provides valuable information. By comparing the color maps of the FWHM for the exposed and non‐exposed regions of the perovskite film (Figure 3c,d), the image related to illuminated perovskite is brighter, which means light causes the CL signals to get broaden. This broadening is expected because light causes ion migration. Interestingly, the domain (grain) boundaries are visible at the FWHM map distribution of the exposed film (Figure 3d). This is due to the contrast between the bandgap of adjacent domains.

The presence of defects in the crystal is necessary for ion migration.^[^ ^10^, ^13^ ^]^ The defects responsible for ion migration are either located inside the crystal (bulk point defects like Schottky defects) or on the surface of the crystal. HIM relies on surface defects to exchange ions between the grains at their interface. These exchanges at the grain boundaries have been shown to be involved in the hysteresis of PSCs.^[^ ^26^ ^]^ In contrast, VIM does not necessarily rely on the crystal surface defects and rather it is more dependent on the point defects within the bulk structure of perovskite.

### 2.3 Passivation Effects on Light‐Induced Ion Migration

**Figure 4:**
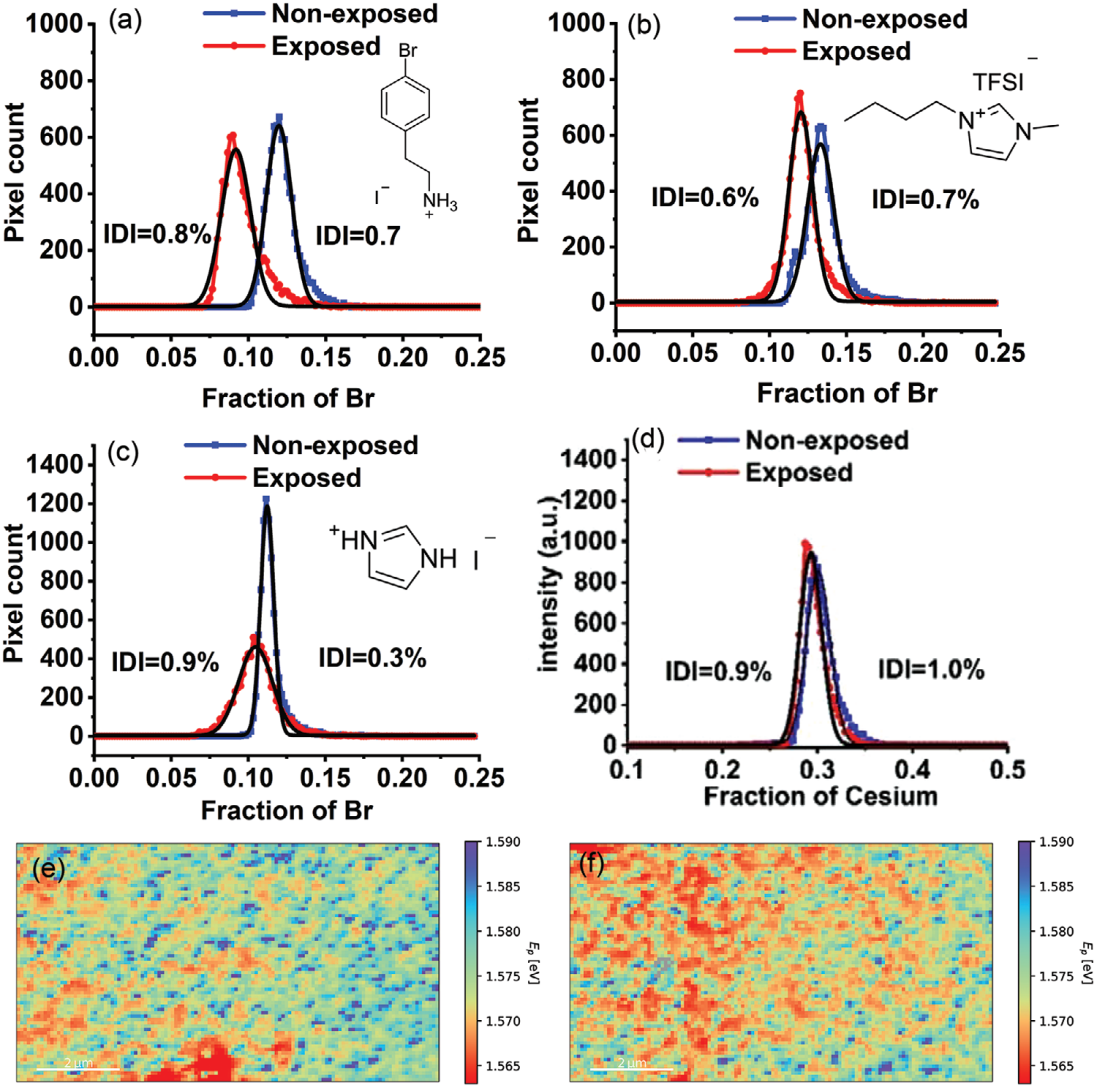
Analysis of the passivated and mixed A‐cation Cs_0.3_FA_0.7_PbI_3_ perovskite films. a) CD histograms for the BrPEAI‐passivated Cs_0.08_MA_0.12_FA_0.80_PbI_2.64_Br_0.36_ film that show IDI of 0.7 ± 0.01% (*n* = 297, Adj. *R* ^2^ = 0.99) for non‐illuminated and 0.8 ± 0.01% (*n* = 297, Adj. *R* ^2^ = 0.96) for the illuminated film. b) BMIM TFSI‐passivated Cs_0.08_MA_0.12_FA_0.80_PbI_2.64_Br_0.36_ film that shows IDI of 0.7 ± 0.01% (*n* = 196, Adj. *R* ^2^ = 0.97) for non‐illuminated and 0.6 ± 0.01% (*n* = 196, Adj. *R* ^2^ = 0.98) for the illuminated film. c) IMI‐passivated Cs_0.08_MA_0.12_FA_0.80_PbI_2.64_Br_0.36_ film that shows IDI of 0.3 ± 0.01% (*n* = 196, Adj. *R* ^2^ = 0.98) for non‐illuminated and 0.9 ± 0.01% (*n* = 196, Adj. *R* ^2^ = 0.99) for the illuminated film. d) CD histograms for Cs_0.3_FA_0.7_PbI_3_ perovskite film that show IDI of 1.0 ± 0.01% (*n* = 196, Adj. *R* ^2^ = 0.98) for non‐illuminated and 0.9 ± 0.02% (*n* = 196, Adj. *R* ^2^ = 0.97) for the illuminated film. The map of fitted peak positions for e) non‐illuminated and f) illuminated Cs_0.3_FA_0.7_PbI_3_ perovskite film (scale bar = 2 µm).

CL was applied to determine whether passivation of the surface defects and/or bulk film takes place and to what extent. Three different passivating agents were evaluated, 4‐bromophenylethylammonium iodide (BrPEAI), which is similar to phenylethylammonium iodide, known to passivate the perovskite surface,^[^ ^27^ ^]^ 1‐butyl‐3‐methylimidazolium bis(trifluoromethylsulfonyl)imide (BMIM TFSI), which is similar to BMIM BF_4_ that is reported to passivate the surface and reduce ion migration,^[^ ^28^ ^]^ with the unstable BF_4_^−^ anion replaced by the stable TFSI^−^ anion. The third compound used was imidazolium iodide (IMI), which based on a work by Alharbi et al., acts to reduce recombination by passivation of perovskite.^[^ ^29^ ^]^ The passivating materials were deposited onto the surface of the perovskite in isopropanol (5 mg mL^−1^) and then annealed for 5 min. Prior to the CL measurements (see the Experimental Section for measurement details), the passivated perovskite films were exposed to light under the same conditions used for the pristine Cs_0.08_MA_0.12_FA_0.80_PbI_2.64_Br_0.36_ film. Analysis of the CL data (**Figure** **4**a) shows that HIM is largely inhibited by the application of BrPEAI (only 0.1% HIM). Additionally, shifts in the CD histogram peaks are observed that may be related to VIM (equivalent to 2.8% Br^−^ difference). The inhibition of HIM is evident by comparing the color maps of fitted peak positions (see Figures S4a and S5a, Supporting Information). Although HIM is mainly inhibited, higher VIM in BrPEAI‐passivated films compared to the non‐passivated Cs_0.08_MA_0.12_FA_0.80_PbI_2.64_Br_0.36_ film (2.8 vs 2%), suggests VIM is an independent process to HIM. A scanning electron microscopy (SEM) image of the BrPEAI‐modified film shows coverage of a layer on top of primary perovskite (see Figure S14, Supporting Information). Based on XRD pattern, the composition of this layer was identified as (BrPEA)_2_PbI_4_ (Figure S15, Supporting Information), with the peaks at 2*θ* ≈5 and 10 corresponding to the 2D perovskite.^[^ ^30^ ^]^

The BMIM TFSI‐passivated film exhibits similar behavior to the BrPEAI‐passivated film. HIM is inhibited (≈−0.1%), but VIM is not inhibited and the difference in Br^−^ content between exposed and non‐exposed films is only 1.3% (see Figure 4b for the CD histogram and Figures S6a and S7a (Supporting Information) for the color maps of the fitted peak positions). The inhibition of HIM by using passivating agents shows that HIM is highly dependent on the surface defects, and presumably, the passivating agent imposes a kinetic barrier which suppresses HIM. This explanation is consistent with the results from McGehee et al., which showed that halide segregation is retarded by an electron‐donating passivating agent.^[^ ^31^ ^]^ As VIM takes place within the boundaries of the perovskite grains it is not inhibited by surface passivation agents. Notably, the mesh‐like pattern, which is an indication of HIM is less pronounced in the FWHM map of the passivated exposed films (Figure S5b, Supporting Information, for BrPEAI‐passivated and Figure S7b, Supporting Information, for BMIM TFSI‐passivated films). However, large cations such as BrPEA^+^ and BMIM^+^ are unable to penetrate into the perovskite structure and fill the inner grains vacancies, and hence they do not suppress VIM. To better show the VIM, CL was performed on the cross section of exposed BMIM TFSI‐passivated films. The data show the surface and top ≈100 nm of the film has a lower bandgap than the rest of the thickness (Figure S16, Supporting Information).

Unlike the passivating agents discussed above, the IMI‐passivated film did not completely inhibit HIM, although it was reduced to 0.6% halide migration compared to the non‐passivated film. Interestingly, VIM was reduced compared to the pristine perovskite film (0.8% vs 2%) (see Figure 4c and Figures S8a and S9a, Supporting Information, for peak position color maps of the IMI‐passivated films). The reduction in VIM may be explained by a study by Zhang et al.,^[^ ^32^ ^]^ which suggests that small imidazolium cations are incorporated into the perovskite lattice, presumably impeding VIM by filling internal defects.

### 2.4 Photoinduced Ion Migration in Cs_0.3_FA_0.7_PbI_3_ Films

Double cation Cs_0.3_FA_0.7_PbI_3_ films were fabricated and exposed to 1 sun white LED light for 12 h under a nitrogen atmosphere and the films were analyzed using CL followed by statistical analysis. Differences in the distribution of fitted peak positions in the PPD histograms between illuminated and non‐illuminated Cs_0.3_FA_0.7_PbI_3_ films must be due to the migration of Cs^+^ and FA^+^ cations. The ratio of the A‐site cations is related to the bandgap in a linear fashion.^[^ ^2^, ^23^ ^]^ Therefore, a similar approach to the Cs_0.08_MA_0.12_FA_0.80_PbI_2.64_Br_0.36_ perovskite was used to convert the PPD to CD histograms. The PPD histograms can be converted to CD histograms using Equation (7)^[33]^

(7) $$\mathrm{Bandgap}\left(\mathrm{eV}\right)=0.3x+1.48$$

where *x* is the fraction of Cs^+^ from the total Cs^+^ and FA^+^ content. The intercept is calculated from the peak position of the CD of the non‐exposed film. Not surprisingly, the lower value of the slope of this formula compared to Equation (1) (0.3 vs 0.62) shows that the changes in the bandgap due to the A‐site cations are less pronounced than the changes in halogen composition.

Interestingly, HIM was not observed for the double cation mixed perovskite whereas VIM was observed (0.7% A‐site cation migration, see Figure 4d–f). The absence of HIM suggests the driving force to segregate A‐cations between the grains is high in the Cs_0.3_FA_0.7_PbI_3_ perovskite. As VIM takes place in Cs_0.3_FA_0.7_PbI_3_ it would appear that A‐site cations, similar to halides, migrate under light illumination. Ionic conduction in lead halide perovskites is considered to be due to halide migration rather than A‐cation migration due to high activation energies for A‐site cation migration.^[^ ^34^ ^]^ However, migration of A‐site cations has been reported in an electrical field for a mixed cation perovskite.^[^ ^35^ ^]^ Light illumination is known to reduce the activation barrier for halide migration and therefore kinetically facilitate photo‐induced segregation.^[^ ^10^, ^36^ ^]^ A‐site cation segregation under light illumination indicates the activation energy for their migration, similar to anions, is lowered. Our results are consistent with prior work by Schelhas et al., which shows the decomposition of FA_0.85_Cs_0.15_PbI_3_ to FAPbI_3_ and CsPbI_3_ under operational conditions.^[^ ^37^ ^]^

### 2.5 Light‐Induced Ion Segregation Mechanisms

**Figure 5:**
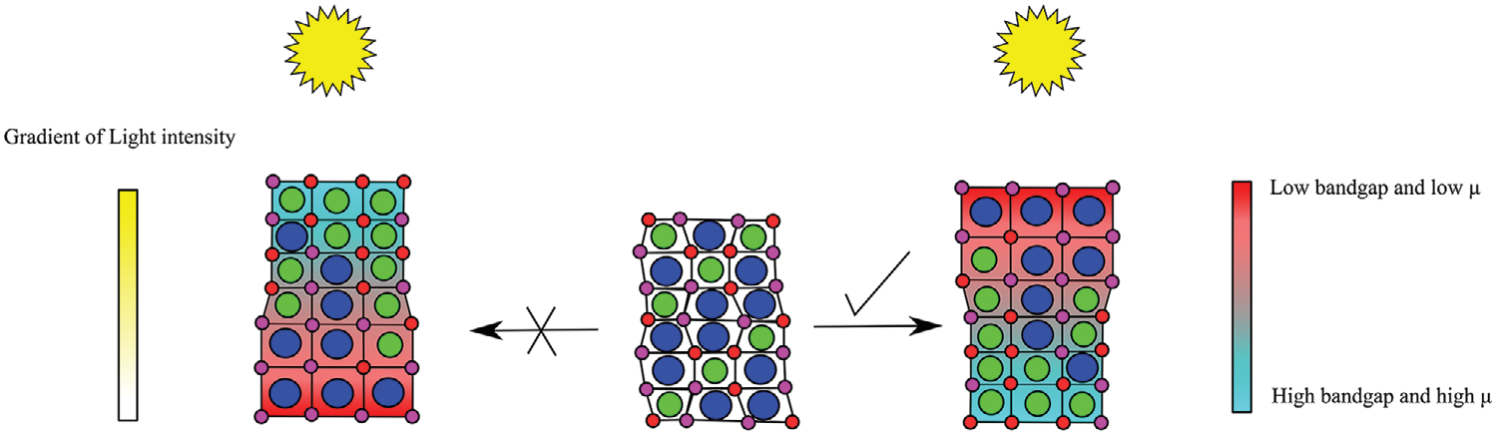
Schematic representation of VIM showing the most stable directionality is which high bandgap perovskite with low shear modulus locates on top which light intensity is the most to achieve lowest light‐induced strain.

Here, we propose a mechanism based on our observations, but it does not mean that other mechanisms and processes cannot take place in such a complex system. It is known that anions and cations in perovskites migrate under light illumination. Ion migration is mediated via surface halide vacancies leading to HIM and via point defects resulting in VIM. VIM has a directionality, which makes the surface always more redshifted. A mechanism based on PL quenching phenomena has also been proposed for light induced‐ion migration mainly in MAPbI_3_ and MAPbI_3−_ *_x_* Cl*_x_* perovskites in which iodide is the mobile component, with the light gradient through the thickness of the film causing an electrical field that leads to migration of iodide ions away from the surface.^[^ ^15^, ^38^ ^]^ However, as iodide is the only mobile component in these studies, this mechanism cannot be used to explain our observations. Based on a study by Walsh et al., the driving force for the segregation is the reduction of the internal strain in alloys of mixed‐lead halide perovskites.^[^ ^11^ ^]^ Moreover, light induces additional strain by unit cell expansion and reduces the barrier for halide migration. Perovskites act to reduce this excessive strain energy by ion segregation processes (HIM and VIM). Therefore, light causes the metastable alloy of perovskite to segregate to reduce the internal strain. Generally, lower strain results in higher PL intensities,^[^ ^39^ ^]^ also our higher CL intensities of exposed films can indicate, the resulting segregated film has lower internal stress (see Figures S2c–S11c, Supporting Information). The VIM is a special case of ion migration in which the ion migration is also dependent on the amount of light that can reach the perovskite, i.e., the interior of perovskite film experiences lower intensities of light than the surface due to the light absorption.^[^ ^13^, ^40^ ^]^ As lead halide‐based perovskites are direct bandgap semiconductors, the light is largely absorbed by the upper levels of the perovskite film, and hence the excessive strain due to light absorption is smaller at the inner region of the film. The light‐induced strain energy (Δ*G*_s_) can be estimated using Equation (8)^[^ ^12^, ^41^ ^]^

(8) $$\Delta G_{s}\left(x,I\right)\approx 4\mu {\delta_{\left(I\right)}}^{2}V$$

where *μ* is shear modulus of the perovskite, *δ* is the fraction of change in the volume to total volume of the perovskite unit cell upon irradiation (with an order of 1%–2% under 1 sun^[^ ^39b^ ^]^), which is a function of light intensity (*I*) and *V* is the molar volume of perovskite. *μ* is dependent on the average Pb—X bond strength and length.^[^ ^42^ ^]^ Therefore, *μ* is larger in Br^−^‐rich perovskites than I^−^‐rich perovskites; moreover, based on density functional theory (DFT) calculations, *μ* is larger for Cs^+^‐rich perovskites than FA^+^‐rich perovskite.^[^ ^43^ ^]^ For the mixed anions or cations perovskites, *μ* is dependent on the molar fraction (*x*) of halides and A‐cations. Low values of *μ* coincide with low bandgap perovskites. In other words, I^−^‐ and FA^+^‐rich perovskites have a lower *μ* (and therefore are more flexible). To minimize Δ*G*_s_, the surface of perovskite which faces the light source and therefore has a higher *δ*, should be composed of perovskite with low values of *μ* (low bandgap) (**Figure** **5**). In this case, perovskites with high values for *μ* (i.e., Br^−^‐ or Cs^+^‐rich) experience lower light intensities and thus their contribution to the overall Δ*G*_s_ value becomes less pronounced. Therefore, physically constraining perovskite grains can inhibit ion migration as shown for CsPb(Br*_x_* I_1−_ *_x_*)_3_ perovskites.^[^ ^12a^ ^]^

If light can help to release the internal strain due to lattice expansion, Elevating temperature should also result in a similar effect, to analyze this, we used CsPb(I_1.5_Br_1.5_) films which has 1:1 ratio of Br^−^ to I^−^ at two different temperatures in the dark for 12 h. This ratio of halides is known to be prone to demixing.^[^ ^4^, ^12^ ^]^ As it is shown in Figure S17 (Supporting Information), the film kept at 12 °C have IDI of 1.6 which is much lower than IDI of the film kept at 37 °C (IDI = 5.8). Interestingly, comparing two films the peak maximum for the film at 37 °C shifted to higher Br content. This could indicate having a redshift in the illuminated films, as explained, is related to the presence of light gradient.

Here, we tried to identify a mechanism that broadly explains both HIM and VIM, but these two processes can have different driving forces. HIM can be explained by trapped polarons that stabilize I^−^‐rich regions that promote the formation of I‐rich clusters.^[^ ^12b^ ^]^ By considering the light gradient through the thickness of the film, polarons can promote more I^−^‐rich regions on the surface which rationalizes VIM.

## 3 Conclusions

A method was developed that allows ion migration to be quantified. Our analysis gives in‐depth information about the nature of light‐induced ion segregation phenomena. Two different processes were observed – vertical migration of ions (VIM) and migration of ions between the grains referred to as HIM). Passivation materials interact with the surface defects, which are responsible for the exchange of ions between the grains and, therefore, inhibit HIM. VIM can be reduced by surface doping with small cations like imidazolium, and big cations cannot influence the inter‐grain defects and therefore do not stop the VIM. Segregation of A‐cations was also observed under light illumination in a vertical direction but not in the horizontal direction. The VIM has a directionality that perovskite with higher bandgap locates face to the light source. Hence, a mechanism of ion migration was proposed invoking halide segregation to reducing the light‐induced strain energy considering the light intensity gradient throughout the film.

## 4 Experimental Section

### Synthesis of BrPEAI

To a cooled (ice water bath) solution of 4‐bromopheylethylamine (516 mg, 2.58 mmol) in acetonitrile (5 mL), 57% hydroiodic acid solution in water (342 µL, 2.58 mmol) was added dropwise. After 0.5 h, the reaction mixture was dried under reduced pressure. Next, the solid residue was dissolved in isopropanol and precipitated via addition of diethyl ether to give a white powder. The resulting powder was recrystallized in isopropanol to give 726 mg of a white powder (86%). ^1^H NMR (400 MHz, DMSO‐*d*_6_) *δ* 7.72 (s, 3H), 7.58–7.19 (m, 4H), 3.04 (t, 2H), 2.83 (t, 2H). ^13^C NMR (101 MHz, DMSO‐*d*_6_) *δ* 137.11, 131.94, 131.53, 120.43, 32.84.

### Synthesis of IMI

To a solution of imidazole (260 mg, 3.8 mmol) in 5 mL acetonitrile, an equivalent quantity of 57% HI solution in water (0.5 mL, 3.8 mmol) was added dropwise. After 0.5 h, the reaction mixture was dried under reduced pressure and the resulting solid was washed with ethyl acetate to give 579 mg of IMI (77.5%). ^1^H NMR (400 MHz, DMF‐*d*_7_) *δ* 9.63 (s, 1H), 8.10 (s, 2H). ^13^C NMR (101 MHz, DMF‐*d*_7_) *δ* 134.78, 119.68.

### Preparation of Precursor Solution for Cs_0.08_MA_0.12_FA_0.80_PbI_2.64_Br_0.36_

A solution of lead iodide (TCI) (548.6 mg, 1.19 mmol), lead bromide (TCI) (57.06 mg, 0.16 mmol), MABr (GreatCell Solar) (17.4 mg, 0.16 mmol), FAI (GreatCell Solar) (178.94 mg, 1.04 mmol), and CsI (GreatCell Solar) (27.02 mg, 0.10 mmol) was prepared in 1 mL mixture of dimethylformamide (DMF, Acros) and dimethyl sulfoxide (DMSO, Acros) (DMF/DMSO = 4:1 volume ratio).

### Preparation of Precursor Solution for IM*_x_* MA_1−_ *_x_* PbI_3_

1.4 m precursor solution of IM*_x_* MA_1−_ *_x_* PbI_3_ (*x* = 5, 10, and 20) was prepared via dissolving lead iodide (645.4 mg, 1.4 mmol) and corresponding quantities of the imidazolium iodide and methylammonium iodide in 1 mL DMSO.

### Preparation of Precursor Solution for CsPbI_1.5_Br_1.5_

1 m precursor solution of CsPbI_1.5_Br_1.5_ was prepared via dissolving lead iodide (115.2 mg, 0.25 mmol), lead bromide (275.3 mg, 0.75 mmol), and CsI (259.8 mg, 1 mmol) in 1 mL DMSO.

### Preparation of Thin Films

100 µL of precursors were deposited on indium doped tin oxide (ITO) via spin‐coating at 6000 rpm for 30 s and annealed for 1 h at 100 °C. The passivating materials were deposited from their solution of 5 mg mL^−1^ in isopropanol on top of perovskite by spin‐coating at 5000 rpm for 30 s followed by a 5 min post‐annealing step at 100 °C.

The cross‐section films were deposited with the same method on a silicon wafer.

### Thin Film Characterization

SEM images were recorded using a high‐resolution scanning electron microscope (Gemini‐SEM 300). An electron beam accelerated to 3 kV was used with either In‐Lens or SE2 detector. The images were measured with the perovskite infiltrated mesoscopic TiO_2_ films supported by FTO glass.

The XRD patterns of the prepared films were measured using a D8 Advance diffractometer from Bruker (Bragg‐Brentano geometry, with an X‐ray tube Cu‐K*α*, *λ* = 1.5406 Å). For regular angular dispersion XRD patterns, the samples were mounted on a reflection spinner sample holder and continually scanned in *θ*–*θ* mode between 5° and 50° 2*θ*. The programmable divergence slit and anti‐scatter slits were set to proper values according to the sample size. The samples were spinning during the data collection with the speed of 2 s circle^−1^. The resulting patterns in XRDML format were converted into (.xy).

### CL Measurement

Prior to the CL measurements the films were exposed to 1 sun illumination white LED light under a N_2_ atmosphere for 12 h at 35 °C. A black mask was used to ensure the temperature homogeneity between exposed and non‐exposed parts of the films. About 10 min after light soaking, samples were mounted in the microscope and after reaching ultra‐high vacuum (after about 1 h), they were cooled to 10 K to avoid mixing of ions during measurements. Spectra were acquired on the Attolight ROSA 4634 CL SEM operating at 3 kV with the sample held at 10 K with a liquid helium cooled holder in a vacuum <10^−7^ Torr. To reduce sample charging, exposed surfaces of the glass substrate were coated with conductive silver paint, and an electric circuit from the film surface to the ground was made. Though the interaction volume at 3 kV has roughly a diameter of 110 nm, Monte Carlo simulations using the freeware program CASINO suggest that 50% of the CL signal emanates from a 20 nm region under the 10 nm SEM electron probe. The electron gun lens current was set to 0.9 A, and objective aperture size of 30 µm was used to improve spatial resolution and limit the current to below 500 pA to reduce electron beam heating and sample degradation. Hyperspectral maps were acquired with 128 pixel × 128 pixel resolution, a pixel size of 77 nm and with a pixel dwell (spectra exposure) time of 80 ms. False‐colored CL maps were subsequently generated by spatially mapping parameters of the model, such as peak FWHM, shift in energy, or integrated intensity. Histograms proceed from the same method. Thereby, the emission of different CL signals associated with the different phases with nanometer precision was spatially identified. Figures below present the resulting maps for each sample.

### Statistical Analysis

The data set presented here consists of 12 hyperspectral maps, both samples exposed to light and kept in the dark, for a total of 89 460 spectra analyzed. For each hyperspectral map, pre‐processing included a step of data cleaning where the CCD spikes were removed. Then, background was averaged between 320 and 340 nm on the wavelength axis (or 3.65 and 3.875 eV), a range where no emission peak was present on any of the samples and subtracted. This ensures that any fluctuation of the CCD baseline during acquisition was well compensated. Then, the wavelength axis was converted to eV scale, paying attention to applying Jacobian conversion on the intensities. Then, a Voigt function model was fitted on the full hyperspectral maps using dedicated fitting ranges. All fitting ranges are located between 1.5 and 2.5 eV or a smaller interval within these bounds. Because the process of fitting these functions is long, it is advantageous to restrict the fit interval when the dispersion of the data set is small. The fit was performed using the nonlinear‐least‐squares algorithm. During the fit, a homoscedastic variance model was used where the variance was evaluated from the difference between the data and a principal component analysis (PCA) model of the data set keeping the most significant 20 components. The number of components was chosen conservatively, so that no physical signal was mistakenly treated as noise, and thus prevent overfitting. Because the FWHM of a Voigt profile has no analytical definition, Olivero and Longbothum's numerical approximation was used for evaluating it.^[^ ^44^ ^]^ This approximation is accurate to a maximal error of 200 ppm. Because maps present some degraded areas, either due to exogeneous impurities in the film or mechanical damages, there are some points on the data sets where the fit procedure fails. This translates to the reduced‐*χ* ^2^ goodness‐of‐fit indicator taking arbitrarily high values and high fit parameters relative errors. The data were filtered by setting thresholds of 500 for reduced‐*χ* ^2^ and more than 10% on the area and peak energy. The same filtering was not applied to the *γ* and *σ* parameters of the Lorentz and Gaussian parts of the Voigt profile, because it happens frequently that one or the other exhibit very large uncertainties in cases where the lineshape is strongly Gaussian, respectively, Lorentzian. This results in fit artifacts where the uncertainty of the FWHM, calculated from the respective uncertainties on *γ*, *σ*, gets arbitrarily large for no reason. In most hyperspectral maps, the points excluded from the analysis represent less than 5% (6% for the BMIM TFSI‐passivated perovskite film). This treatment yielded data sets ranging from 8410 to 8946 points for performing CD histogram analyses, exported as ascii files. All the hyperspectral pre‐treatment, including model fitting, was performed with the open‐source python library hyperspy (https://hyperspy.org).

CD histograms were fitted with a built‐in fitting program of OriginPro 2018 (GaussAmp). The fitting parameters are presented in Table S1 (Supporting Information).

CL maps extracted from fit parameters were always represented using the same normalization of the color scale, both for the illuminated and non‐illuminated samples. This resulted in improved eye‐to‐eye comparison between data sets.

The data sets used in the CD histogram range from 8410 to 8946 points depending on the number of the points from hyperspectral ignored due to having a bad fit (showed in gray in the images).

## Conflict of Interest

The authors declare no conflict of interest.

## Supporting information

Supporting Information

## Data Availability

The data that support the findings of this study are openly available in figshare at https://doi.org/10.6084/m9.figshare.17127431, reference number 17127431.
