# Supplementary material for: Deconvolution of Light‐Induced Ion Migration Phenomena by Statistical Analysis of Cathodoluminescence in Lead Halide‐Based Perovskites
Source: Adv Sci (Weinh). 2022 Mar 3;9(13):2103729. doi: 10.1002/advs.202103729 (PMC9069390; doi:10.1002/advs.202103729)
Supplement: Supplementary file 1 — Supporting Information [file ADVS-9-2103729-s001.pdf]

Supporting Information

Deconvolution of Light-induced Ion Migration Phenomena by Statistical Analysis of Cathodoluminescence in Lead Halide-based Perovskites

Erfan Shirzadi, Nicolas Tappy, Fatemeh Ansari, Mohammad Khaja Nazeeruddin,[*] Anders Hagfeldt,[*] and Paul J. Dyson[*]

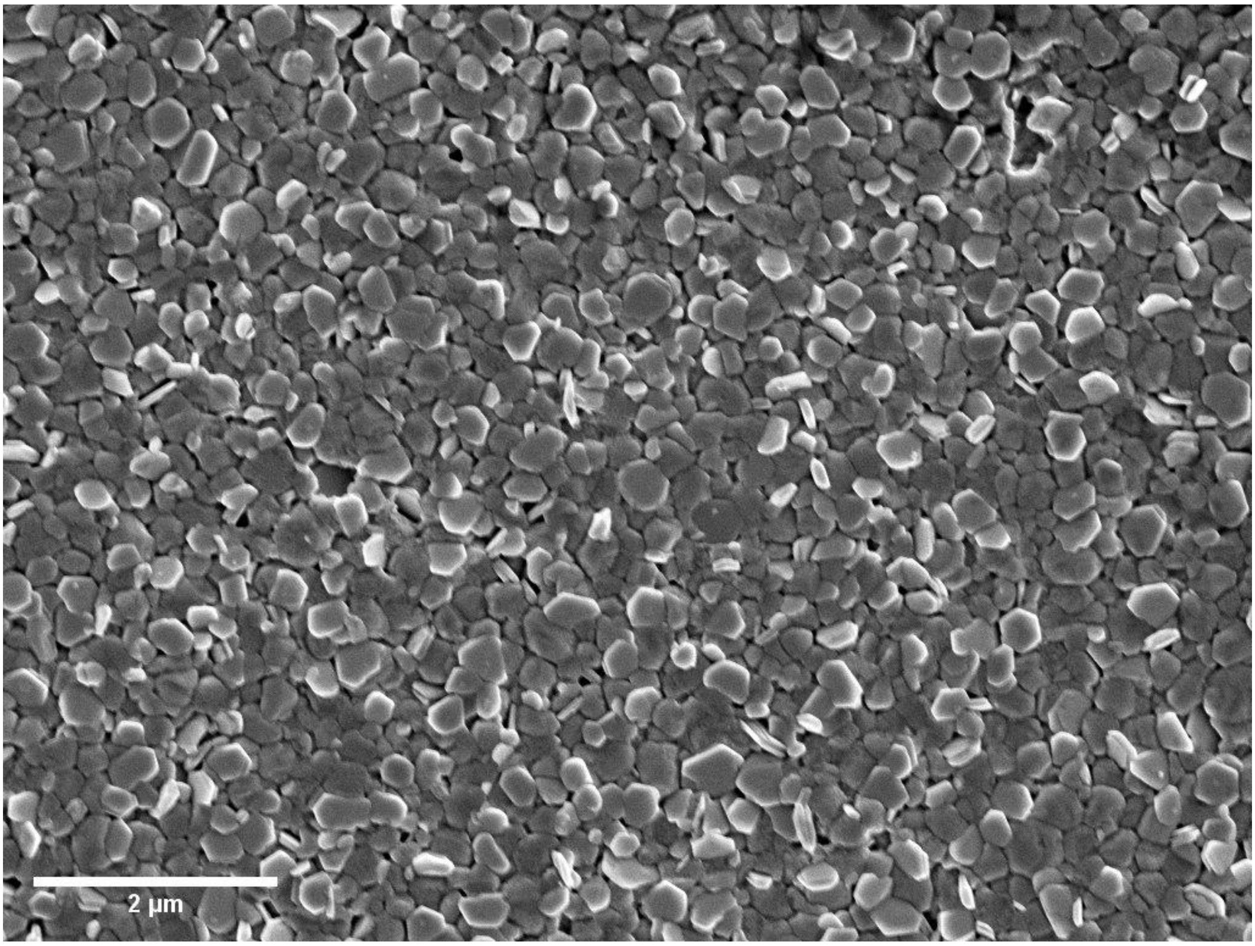

Figure S1. The secondary electron image of $Cs_{0.08}MA_{0.12}FA_{0.80}PbI_{2.64}Br_{0.36}$ film.

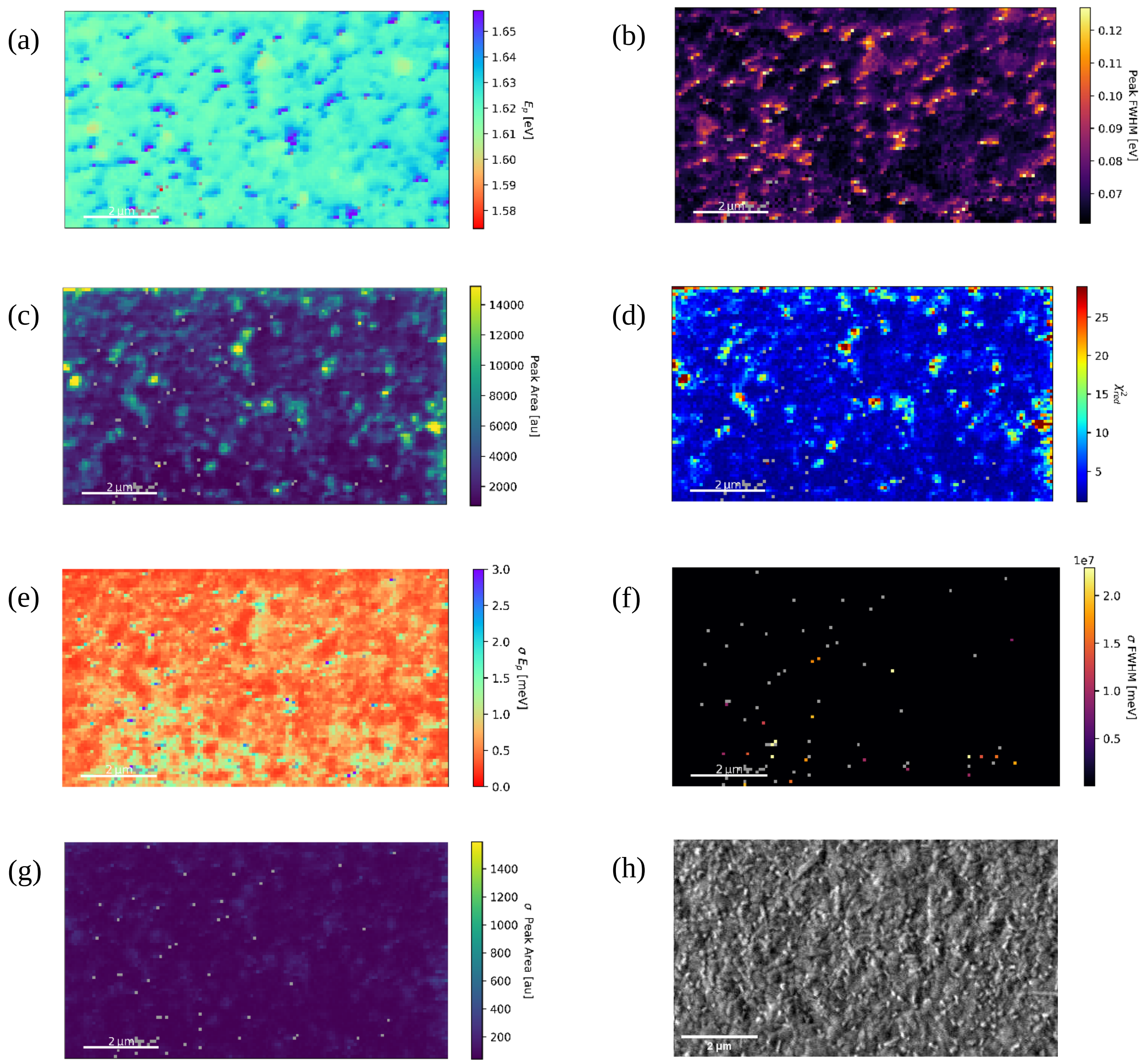

Figure S2. Map of (a) fitted peak centers (b) FWHM of fitted peaks (c) the emission peak area (d) goodness of the fit (e) uncertainty map of the fitted peak centers, (f) uncertainty of fitted peak areas, (g) uncertainty of FWHM of the fitted areas of non-illuminated pristine $Cs_{0.08}MA_{0.12}FA_{0.80}PbI_{2.64}Br_{0.36}$ film. h) secondary electron image of the studied region.

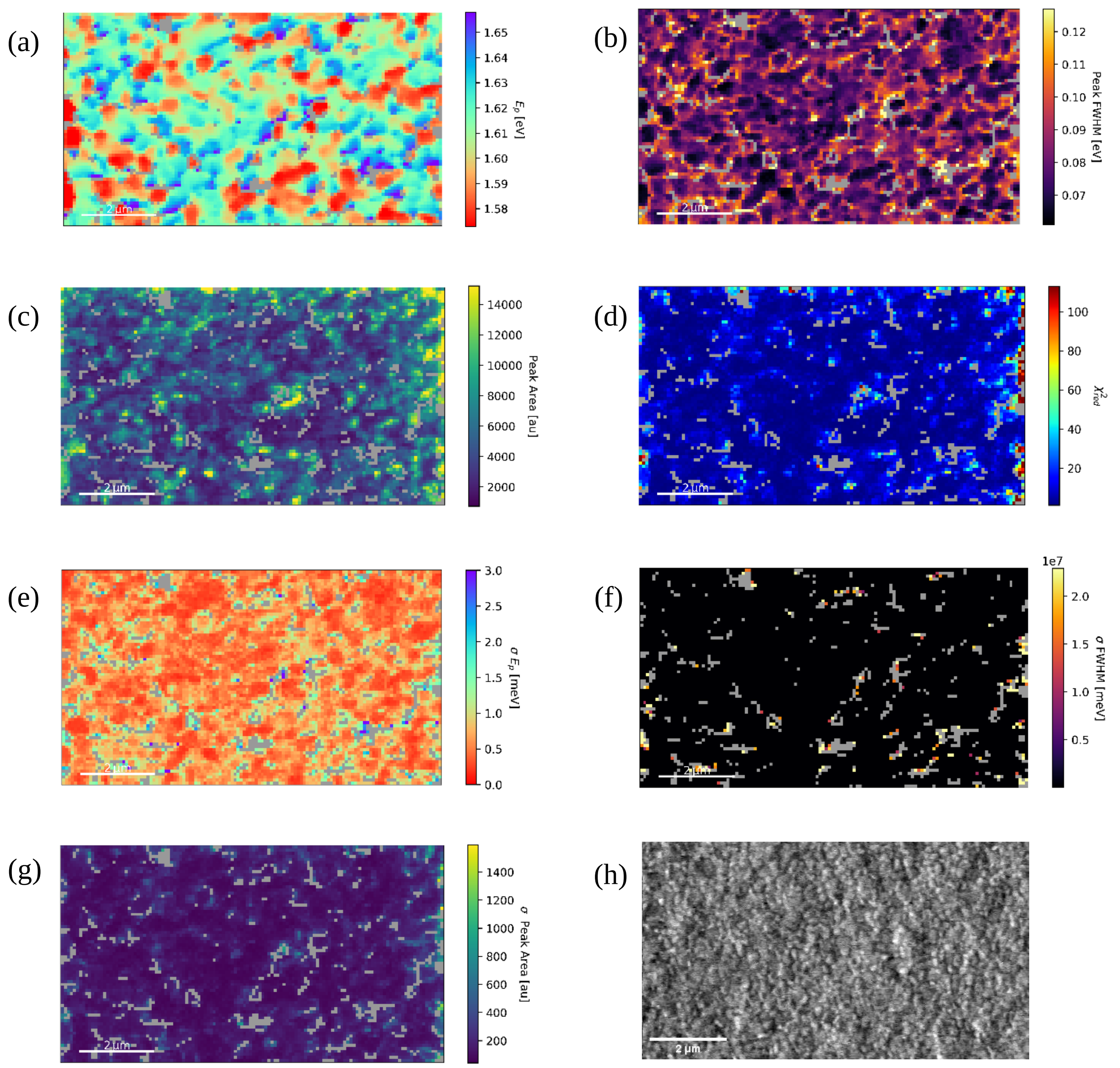

Figure S3. Map of (a) fitted peak centers (b) FWHM of fitted peaks (c) the emission peak area (d) goodness of the fit (e) uncertainty map of the fitted peak centers, (f) uncertainty of fitted peak areas, (g) uncertainty of FWHM of the fitted areas of illuminated pristine $Cs_{0.08}MA_{0.12}FA_{0.80}PbI_{2.64}Br_{0.36}$ film. h) secondary electron image of the studied region.

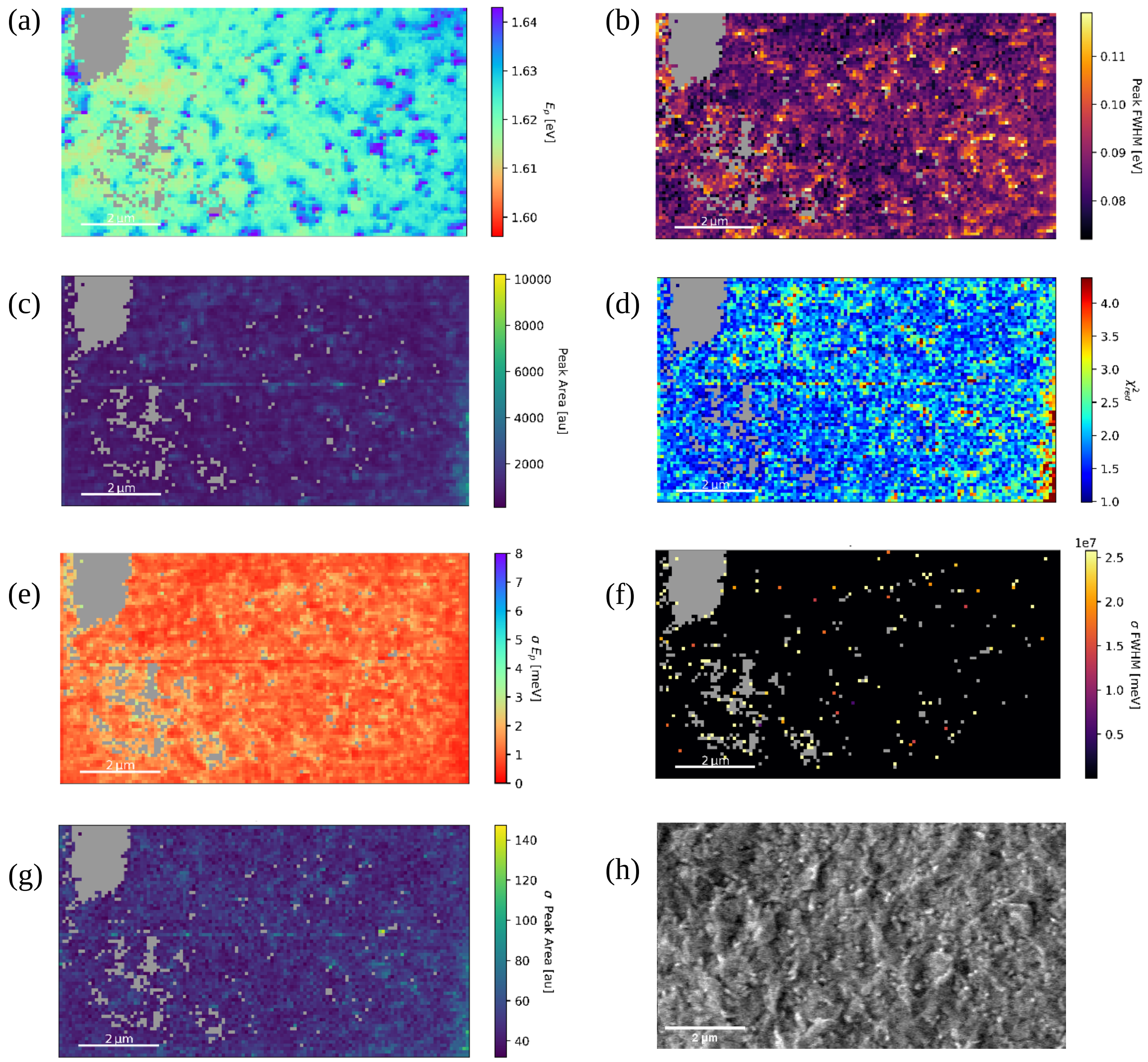

Figure S4. Map of (a) fitted peak centers (b) FWHM of fitted peaks (c) the emission peak area (d) goodness of the fit (e) uncertainty map of the fitted peak centers, (f) uncertainty of fitted peak areas, (g) uncertainty of FWHM of the fitted areas of non-illuminated BrPEAI-passivated perovskite film. h) secondary electron image of the studied region.

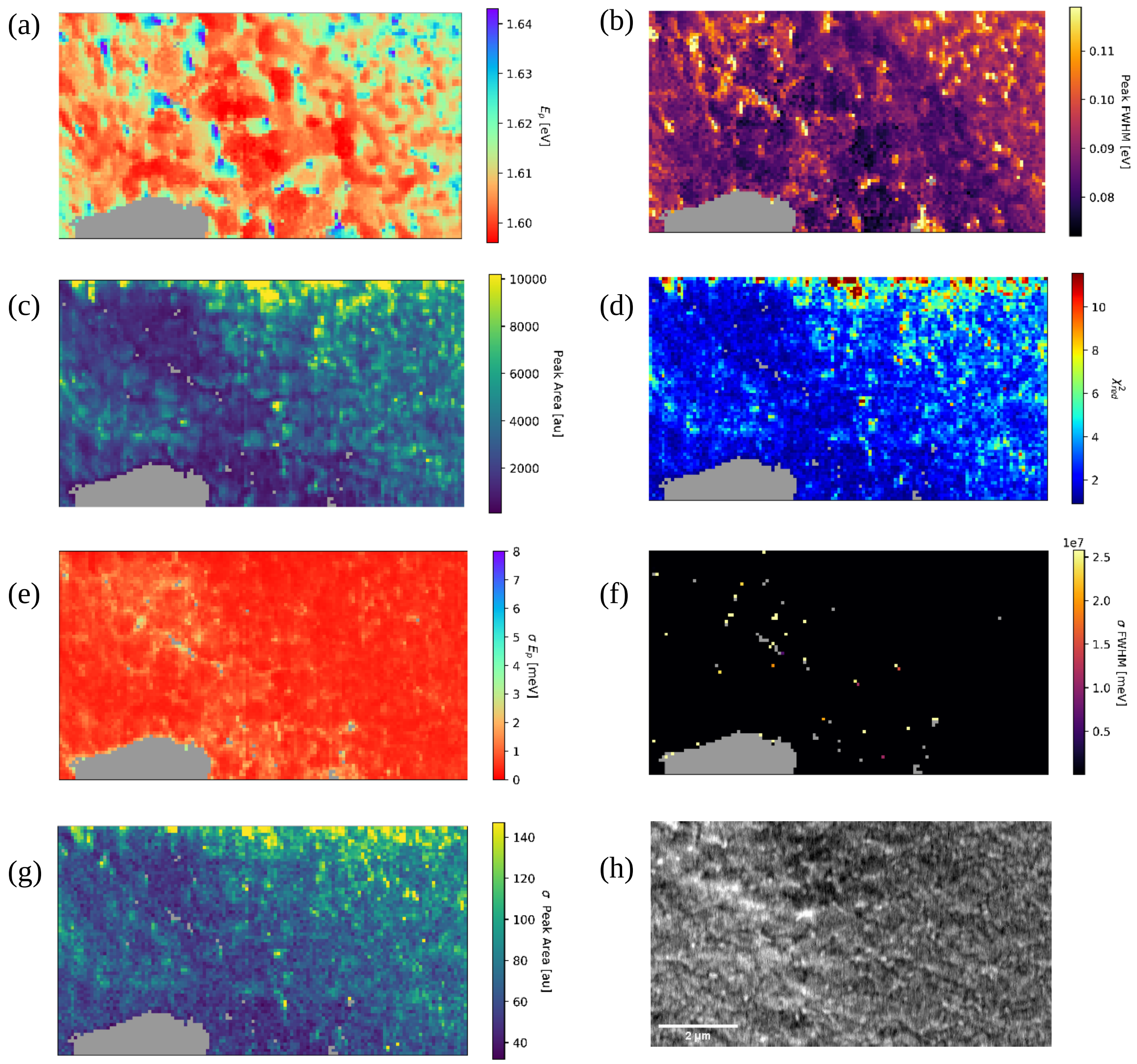

Figure S5. Map of (a) fitted peak centers (b) FWHM of fitted peaks (c) the emission peak area (d) goodness of the fit (e) uncertainty map of the fitted peak centers, (f) uncertainty of fitted peak areas, (g) uncertainty of FWHM of the fitted areas of illuminated BrPEAI-passivated perovskite film. h) secondary electron image of the studied region.

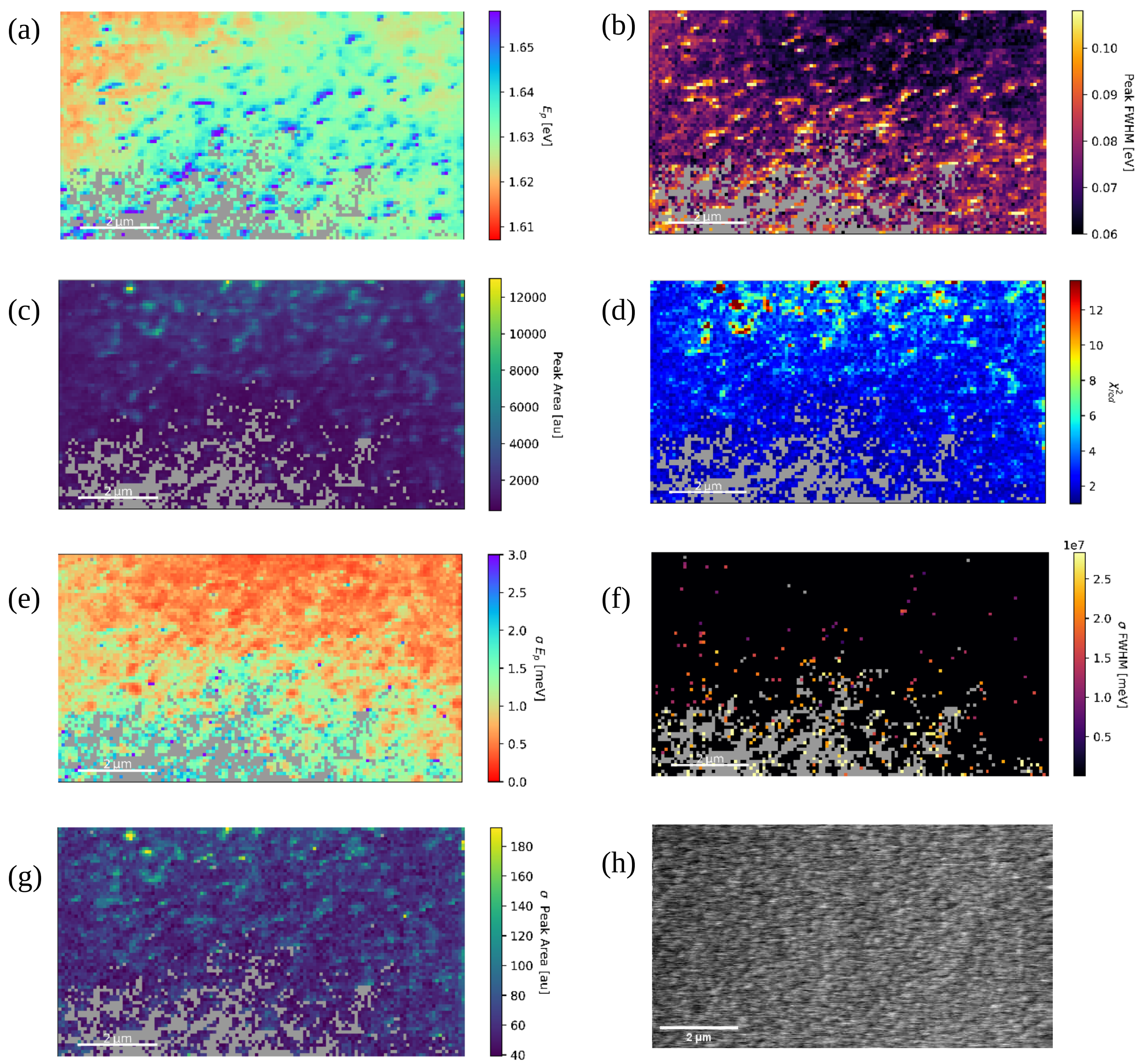

Figure S6. Map of (a) fitted peak centers (b) FWHM of fitted peaks (c) the emission peak area (d) goodness of the fit (e) uncertainty map of the fitted peak centers, (f) uncertainty of fitted peak areas, (g) uncertainty of FWHM of the fitted areas of non-illuminated BMIM TFSI-passivated perovskite film. h) secondary electron image of the studied region.

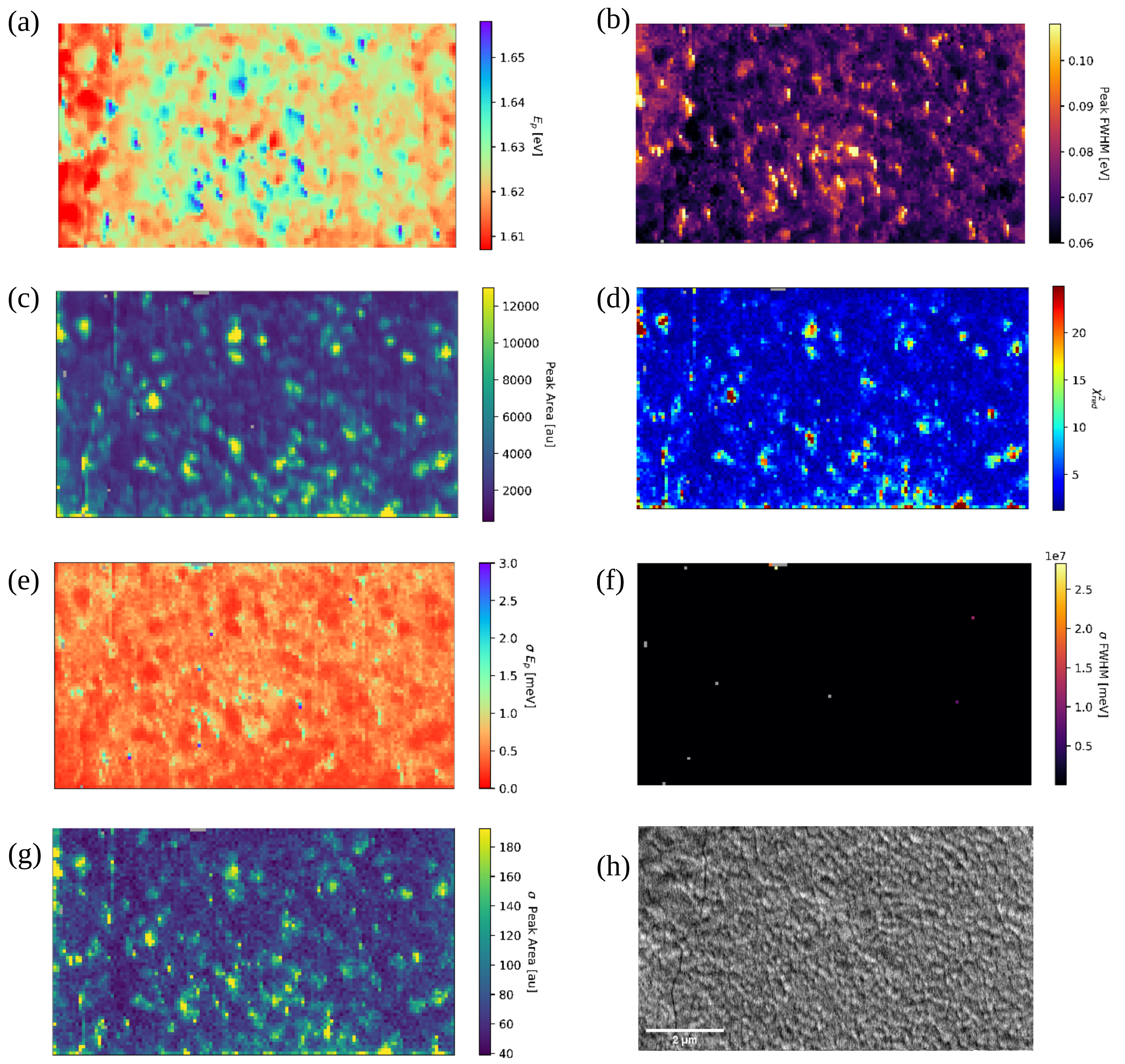

Figure S7. Map of (a) fitted peak centers (b) FWHM of fitted peaks (c) the emission peak area (d) goodness of the fit (e) uncertainty map of the fitted peak centers, (f) uncertainty of fitted peak areas, (g) uncertainty of FWHM of the fitted areas of illuminated BMIM TFSI-passivated perovskite film. h) secondary electron image of the studied region.

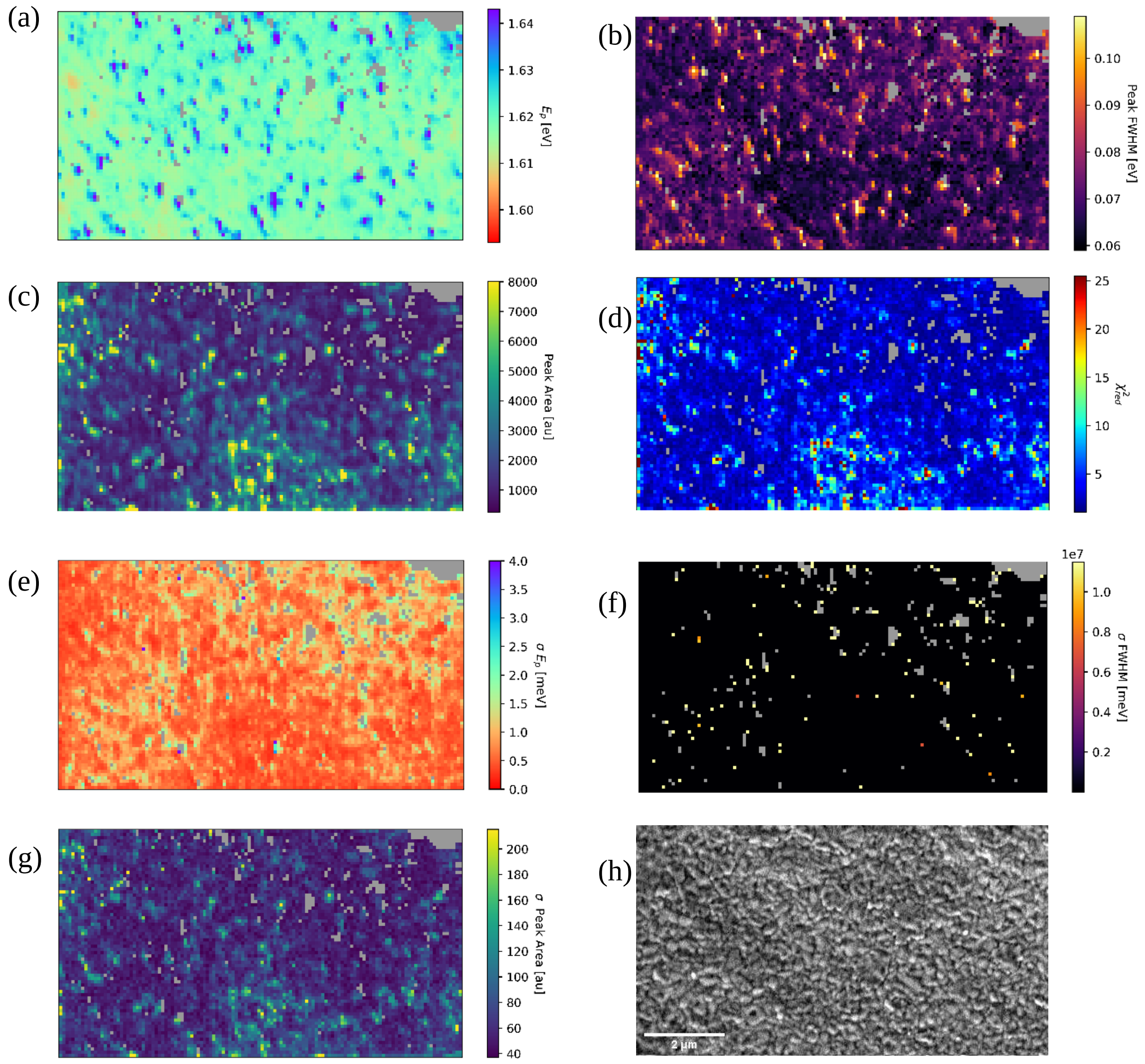

Figure S8. Map of (a) fitted peak centers (b) FWHM of fitted peaks (c) the emission peak area (d) goodness of the fit (e) uncertainty map of the fitted peak centers, (f) uncertainty of fitted peak areas, (g) uncertainty of FWHM of the fitted areas of non-illuminated IMI-passivated perovskite film. h) secondary electron image of the studied region.

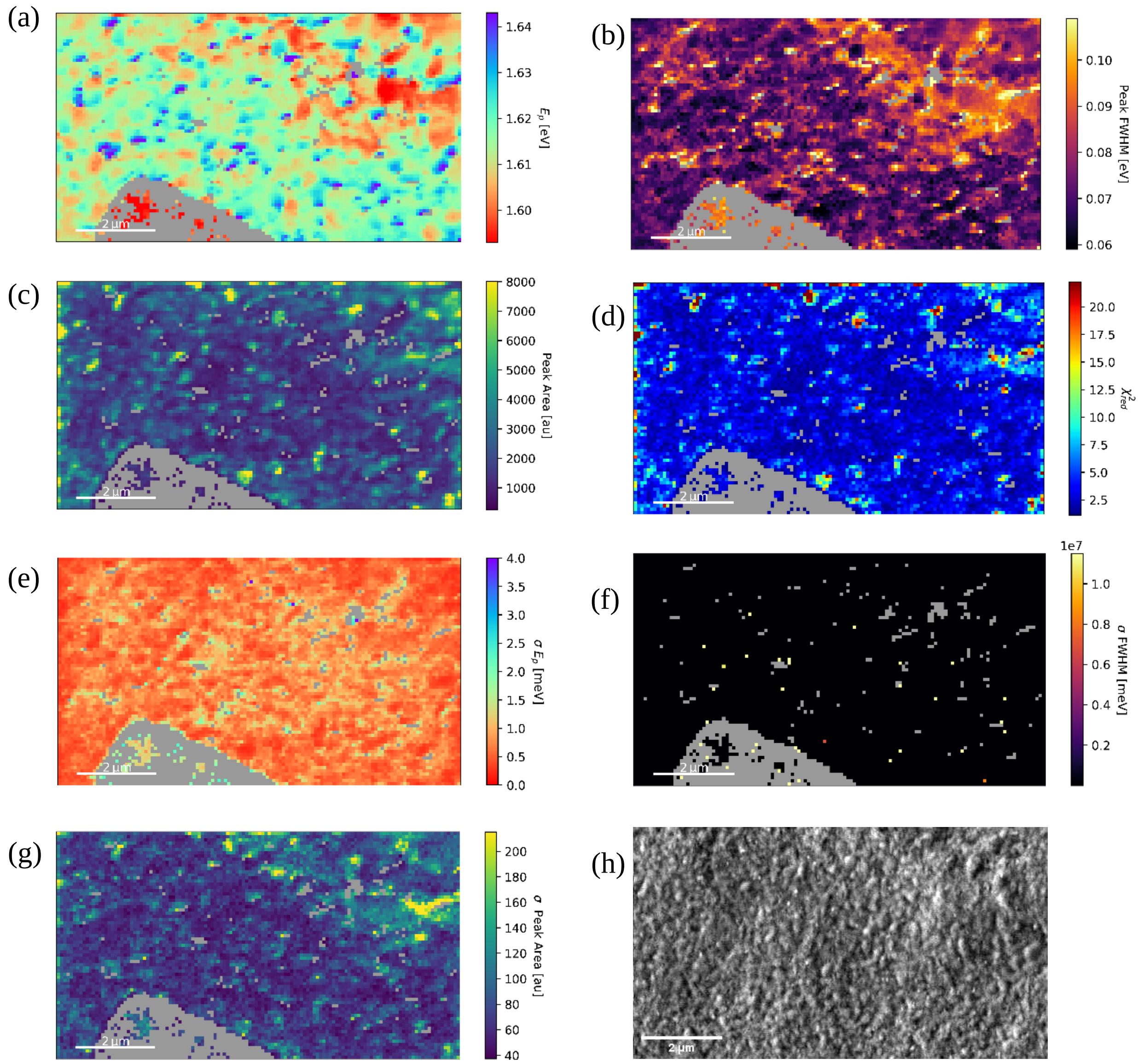

Figure S9. Map of (a) fitted peak centers (b) FWHM of fitted peaks (c) the emission peak area (d) goodness of the fit (e) uncertainty map of the fitted peak centers, (f) uncertainty of fitted peak areas, (g) uncertainty of FWHM of the fitted areas of illuminated IMI-passivated perovskite film. h) secondary electron image of the studied region.

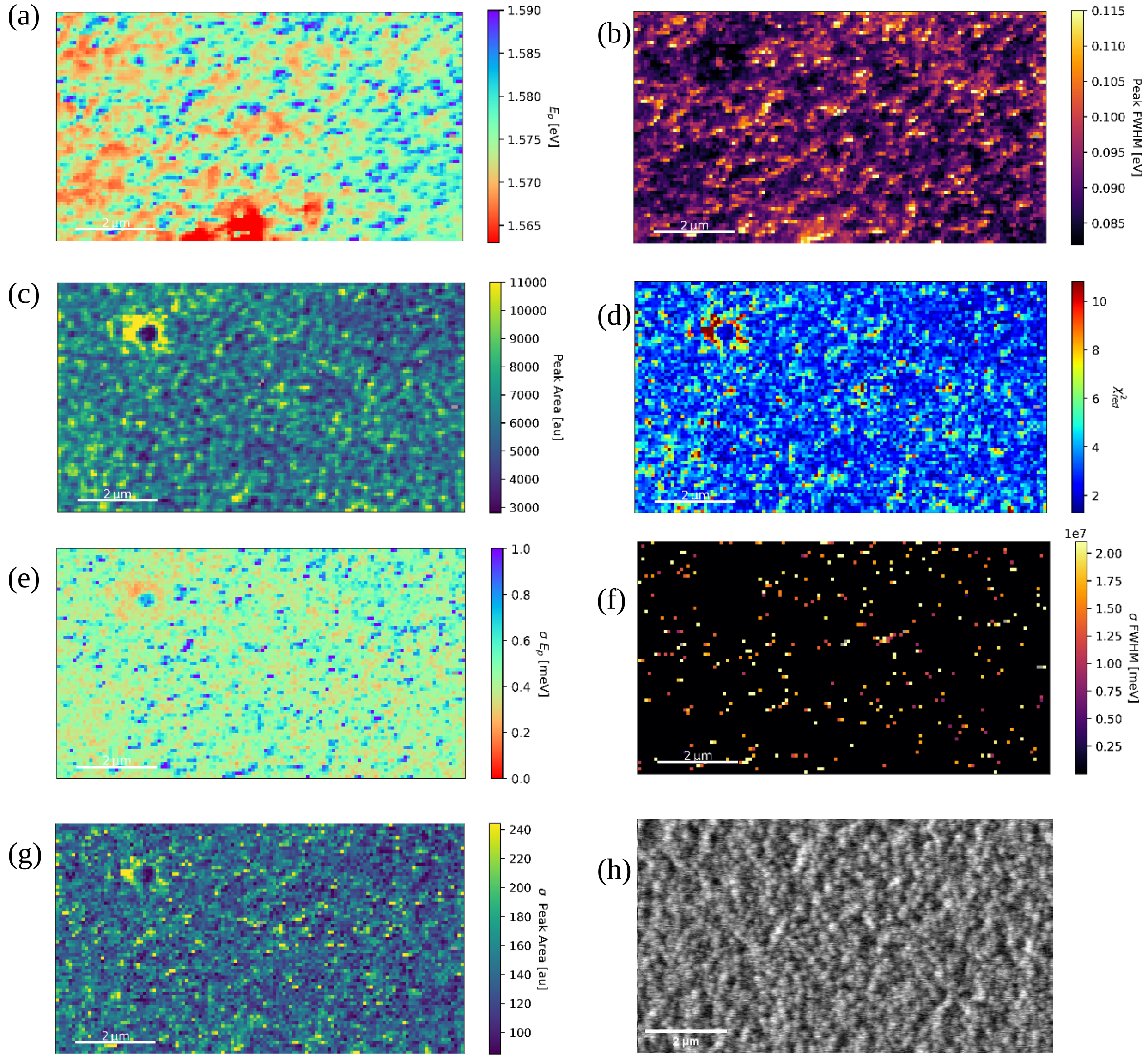

Figure S10. Map of (a) fitted peak centers (b) FWHM of fitted peaks (c) the emission peak area (d) goodness of the fit (e) uncertainty map of the fitted peak centers, (f) uncertainty of fitted peak areas, (g) uncertainty of FWHM of the fitted areas of non-illuminated $Cs_{0.3}FA_{0.7}PbI_3$ perovskite film. h) secondary electron image of the studied region.

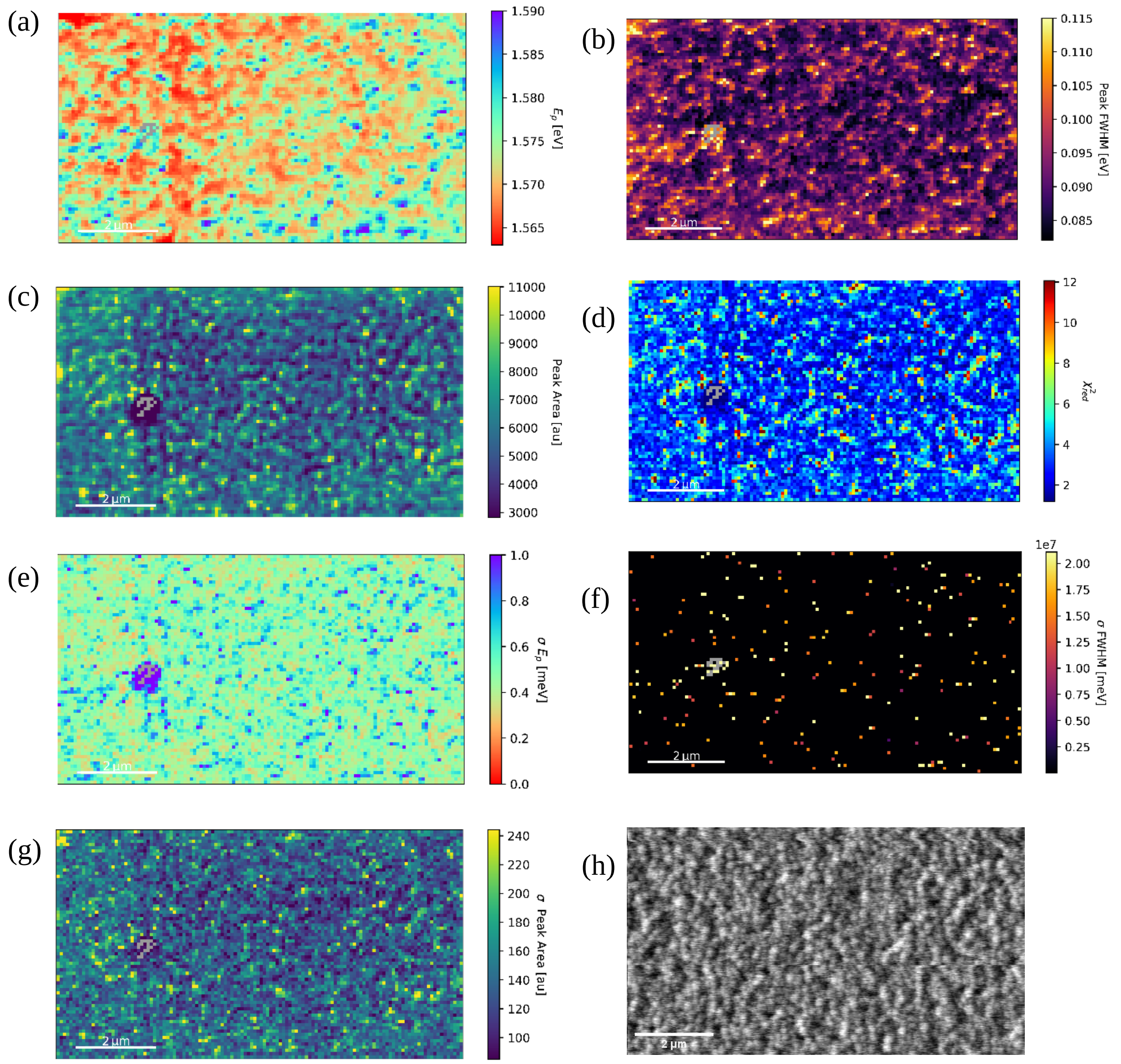

Figure S11. Map of (a) fitted peak centers (b) FWHM of fitted peaks (c) the emission peak area (d) goodness of the fit (e) uncertainty map of the fitted peak centers, (f) uncertainty of fitted peak areas, (g) uncertainty of FWHM of the fitted areas of illuminated $Cs_{0.3}FA_{0.7}PbI_3$ perovskite film. h) secondary electron image of the studied region.

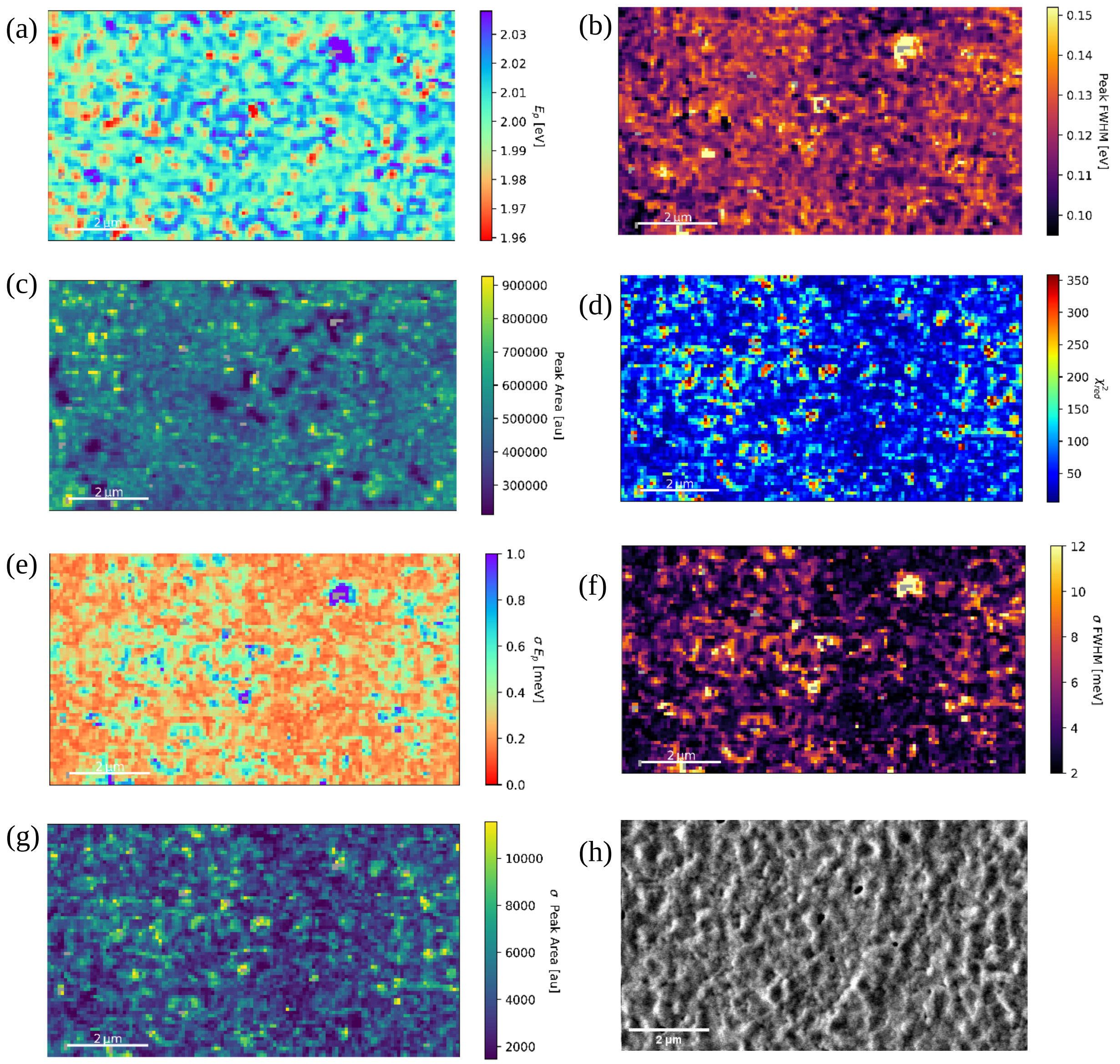

Figure S12. Map of (a) fitted peak centers (b) FWHM of fitted peaks (c) the emission peak area (d) goodness of the fit (e) uncertainty map of the fitted peak centers, (f) uncertainty of fitted peak areas, (g) uncertainty of FWHM of the fitted areas of non-illuminated $CsPbI_{1.5}Br_{1.5}$ perovskite film kept at 12°C. (h) secondary electron image of the studied region.

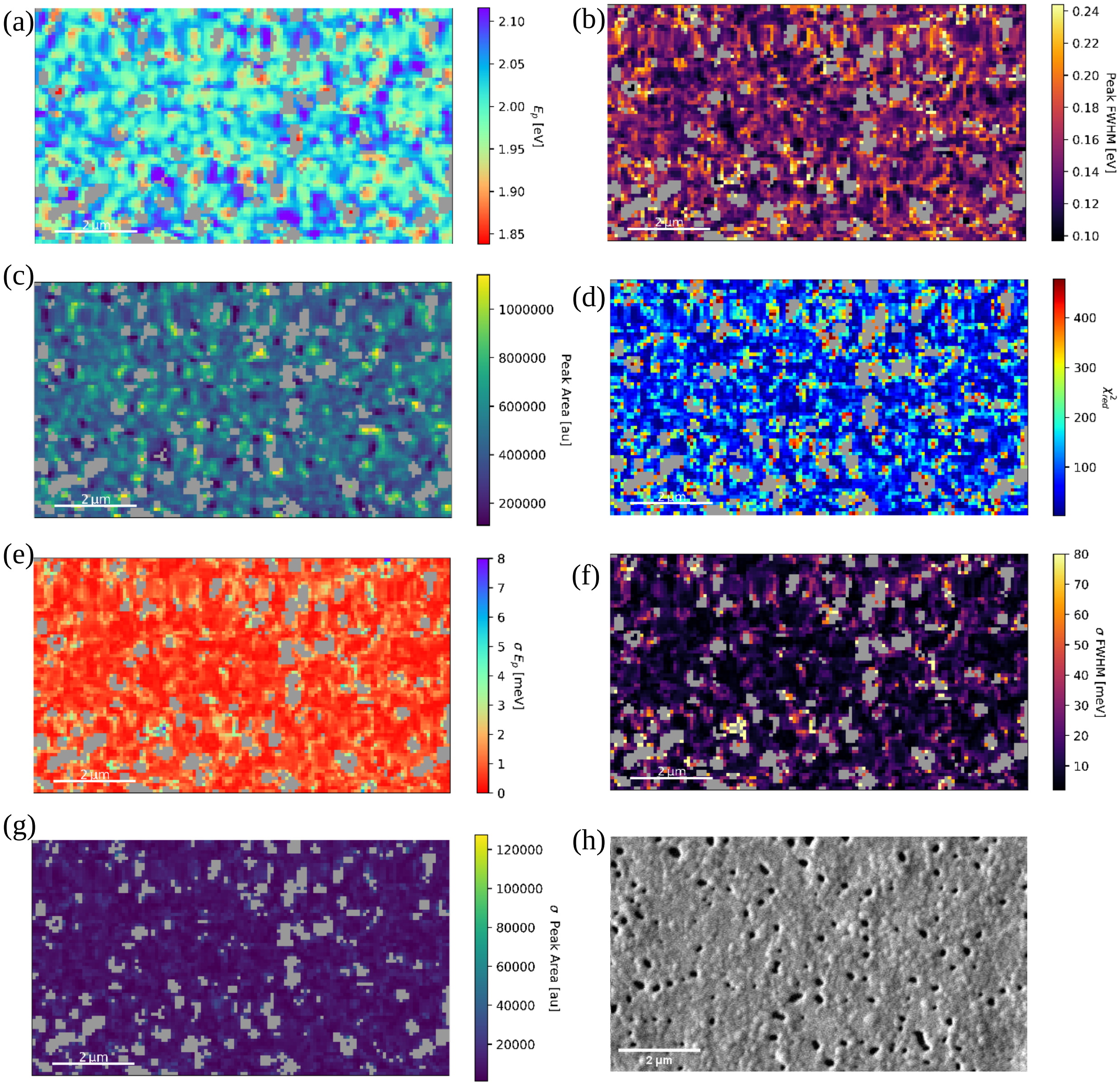

Figure S13. Map of (a) fitted peak centers (b) FWHM of fitted peaks (c) the emission peak area (d) goodness of the fit (e) uncertainty map of the fitted peak centers, (f) uncertainty of fitted peak areas, (g) uncertainty of FWHM of the fitted areas of non-illuminated $CsPbI_{1.5}Br_{1.5}$ perovskite film kept at 37°C. (h) secondary electron image of the studied region.

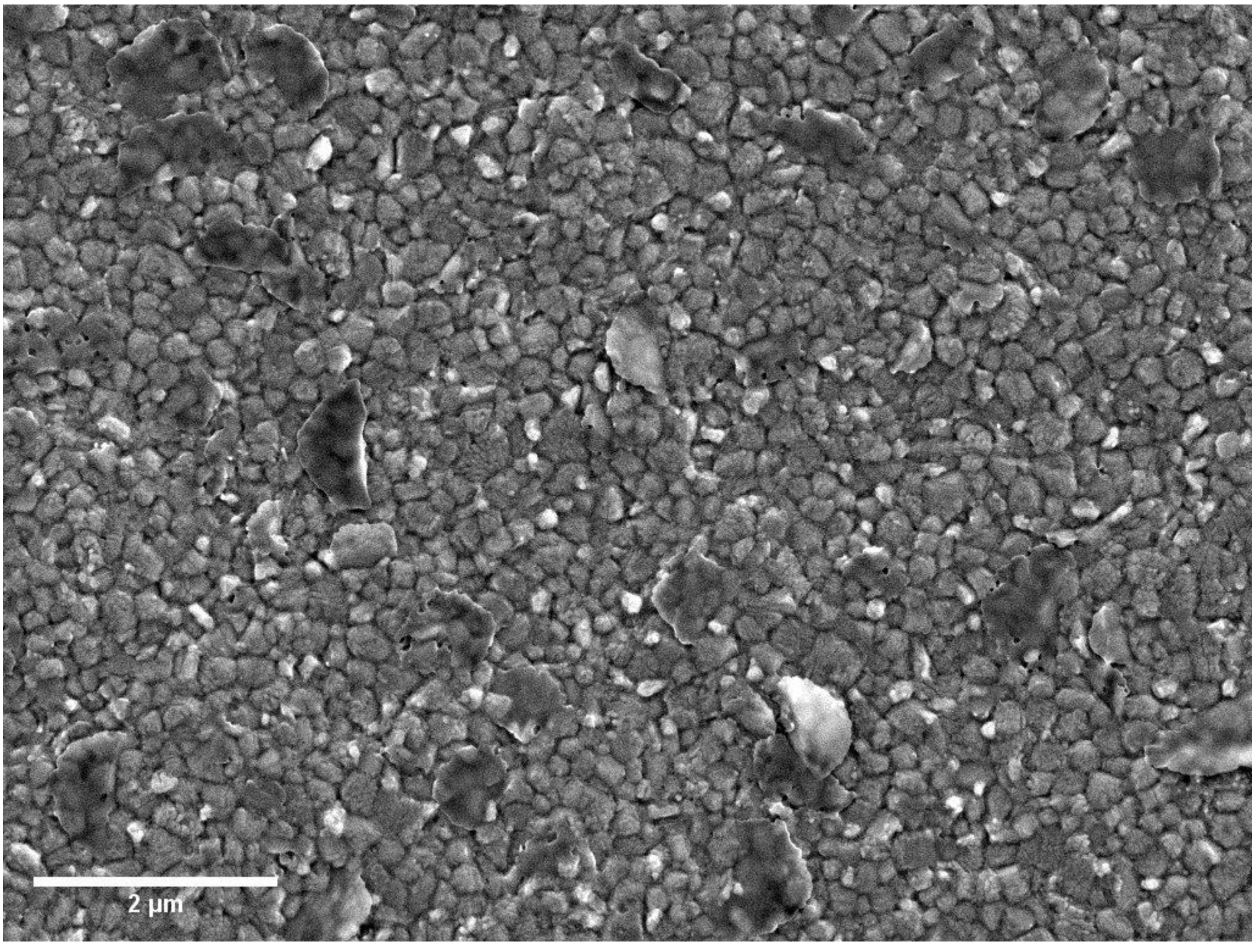

Figure S14. SEM image of BrPEAI-passivated film that shows coverage of surface with BrPEAI-salt.

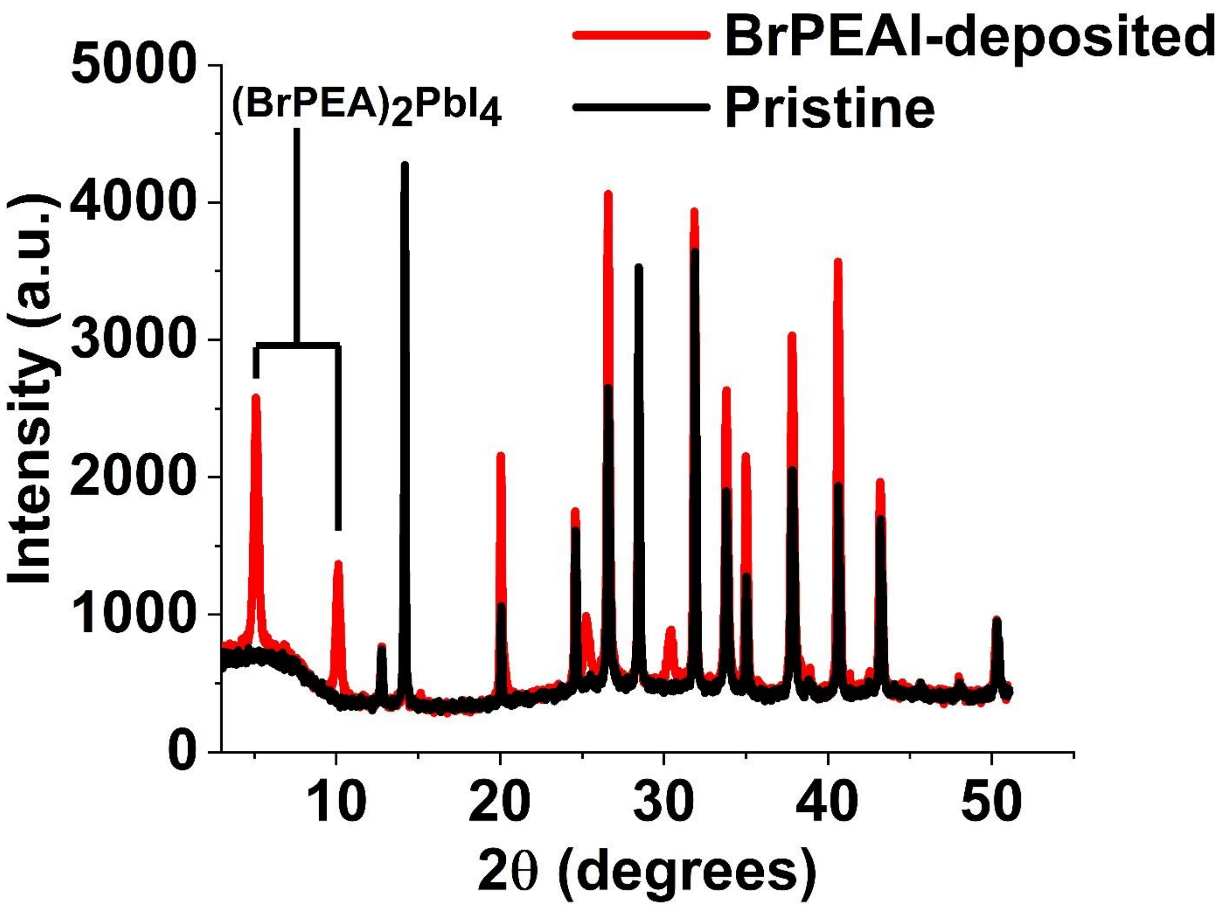

Figure S15. The XRD pattern of BrPEAI-deposited vs pristine film.

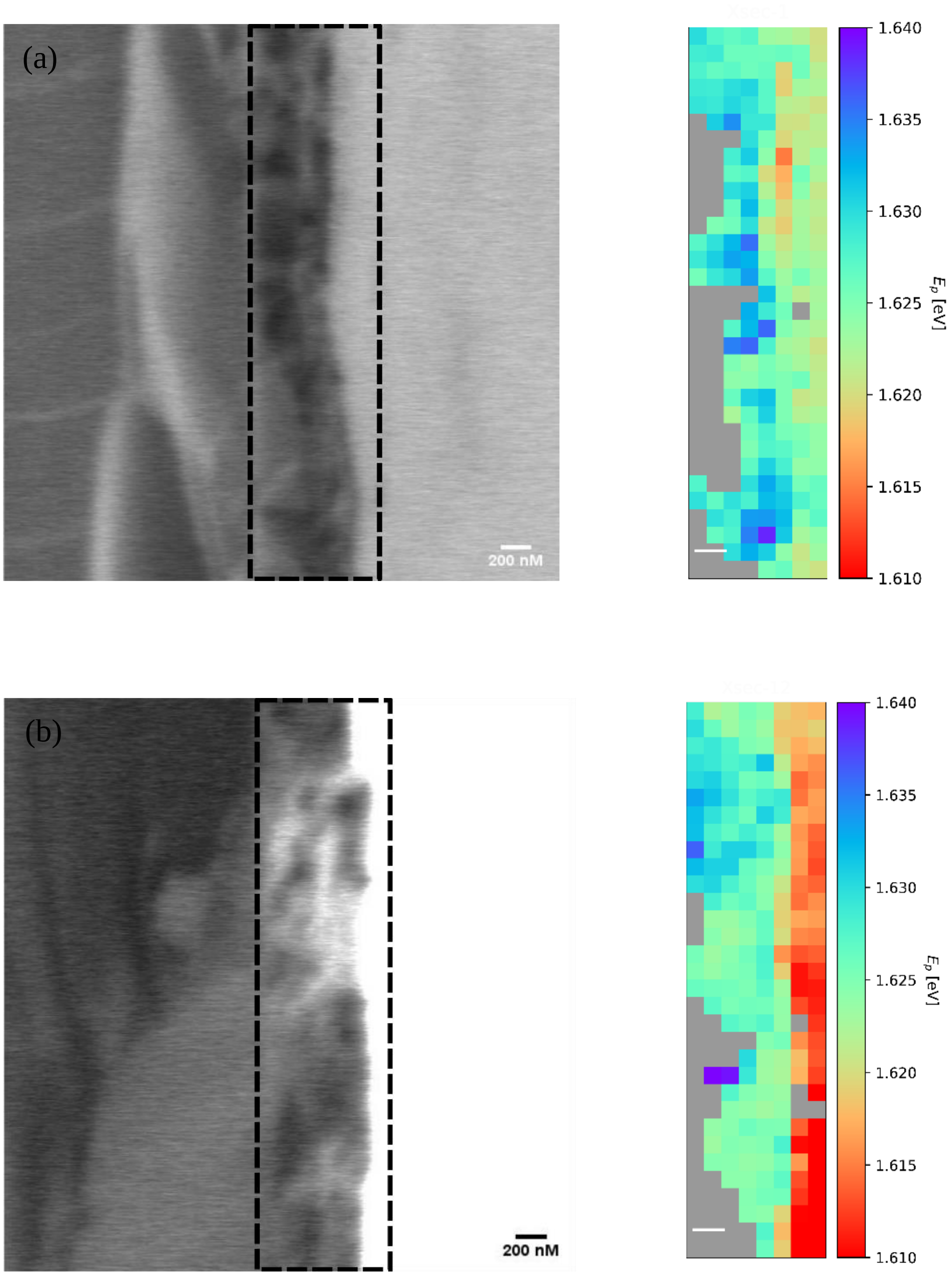

Figure S16. Cross section image and emission map of (a) non-illuminated and (b) illuminated BMIM TFSI passivated films.

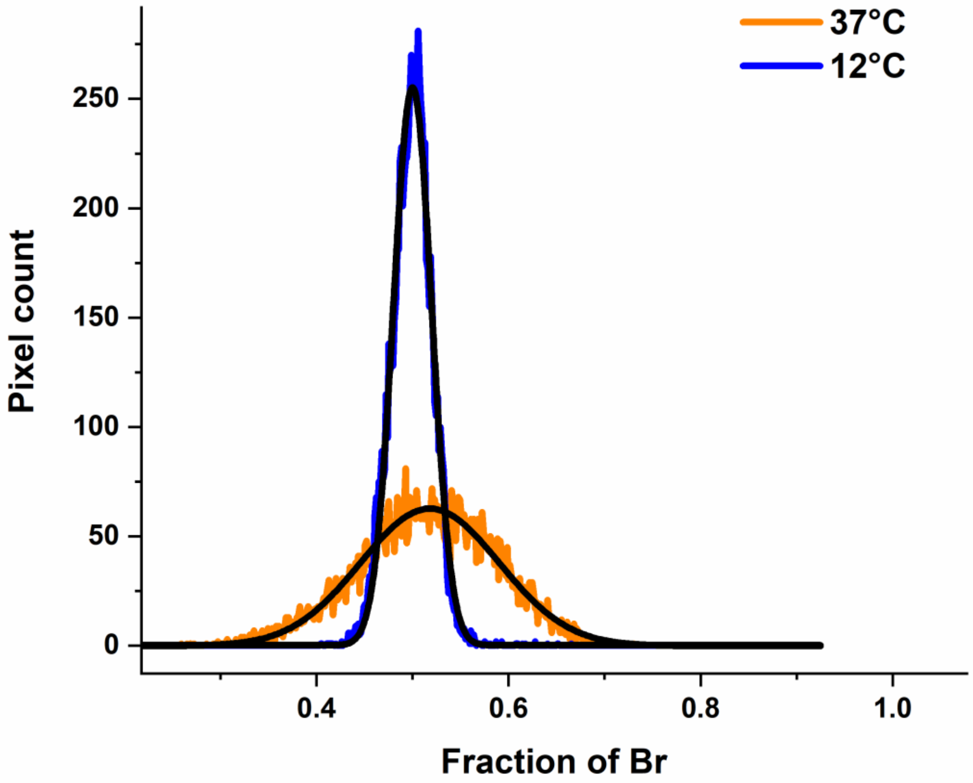

Figure S17. CD histograms for the $CsPbI_{1.5}Br_{1.5}$ for two different temperatures that shows IDI of 1.6±0.01% (n=596, Adj. R^2=0.99) for the film kept at 12°C and 5.8±0.1% (n=569, Adj. R^2=0.97) for the film kept at 37°C for 12h.

Table S1. The CD graph Gaussian fit parameters with $y=y_0+A\exp(-0.5((x-x_c)/w)^2)$ formula.

Film	y_0	x_c	w	A	FWHM	Adj. R^2	n
Pristine, non-illuminated	5.7±2.0	0.12±1.1E-4	0.0063±1.1E-4	814±12.5	0.015	0.97	196
Pristine, illuminated	-2.1±2.3	0.10±6.0E-4	0.035±7.2E-4	164.7±2.6	0.082	0.96	196
BrPEAI-passivated, non-illuminated	1.4±0.8	0.12±7.6E-3	0.0082±7.7E-5	640.4±5.2	0.019	0.99	297
BrPEAI-passivated, illuminated	2.4±1.3	0.092±1.6E-4	0.0094±1.6E-4	554.5±8.1	0.021	0.96	297
BMIM TFSI-passivated, non-illuminated	3.7±1.5	0.13±1.4E-4	0.0085±1.4E-4	565.4±7.9	0.020	0.97	196
BMIM TFSI-passivated, illuminated	5.3±1.4	0.12±1.0E-4	0.0077±1.1E-4	677.9±7.9	0.018	0.98	196
IMI-passivated, non-illuminated	5.2±1.8	0.11±5.7E-4	0.0043±5.8E-5	1183.4±14	0.010	0.98	196
IMI-passivated, illuminated	2.8±0.9	0.105±1.1E-4	0.011±1.1E-4	458.0±4.1	0.026	0.99	196
$Cs_{0.3}FA_{0.7}PbI_3$, non-illuminated	3.0±1.7	0.30±1.8E-4	0.013±1.8E-4	854.2±10.6	0.030	0.98	196
$Cs_{0.3}FA_{0.7}PbI_3$, illuminated	2.5±2.0	0.29±1.8E-4	0.012±1.9E-4	939.8±13.0	0.027	0.97	196
$CsPbI_{1.5}Br_{1.5}$ @12°C	0.2	0.50±1.1E-4	0.020±1.1E-4	255.1±1.2	0.04695	0.99	596
$CsPbI_{1.5}Br_{1.5}$ @37°C	-0.1±0.2	0.52±6.3E-4	0.072±7.1E-4	62.8±0.49	0.17	0.97	596
